# Entrapment of Viral Capsids in Nuclear PML Cages Is an Intrinsic Antiviral Host Defense against Varicella-Zoster Virus

**DOI:** 10.1371/journal.ppat.1001266

**Published:** 2011-02-03

**Authors:** Mike Reichelt, Li Wang, Marvin Sommer, John Perrino, Adel M. Nour, Nandini Sen, Armin Baiker, Leigh Zerboni, Ann M. Arvin

**Affiliations:** 1 Departments of Pediatrics and Microbiology & Immunology, Stanford University School of Medicine, Stanford, California, United States of America; 2 Cell Sciences Imaging Facility, Stanford University School of Medicine, Stanford, California, United States of America; 3 Department of Virology, Max von Pettenkofer-Institute, Ludwig-Maximilians-University, Munich, Germany; MRC Virology Unit Glasgow, United Kingdom

## Abstract

The herpesviruses, like most other DNA viruses, replicate in the host cell nucleus. Subnuclear domains known as promyelocytic leukemia protein nuclear bodies (PML-NBs), or ND10 bodies, have been implicated in restricting early herpesviral gene expression. These viruses have evolved countermeasures to disperse PML-NBs, as shown in cells infected *in vitro,* but information about the fate of PML-NBs and their functions in herpesvirus infected cells *in vivo* is limited. Varicella-zoster virus (VZV) is an alphaherpesvirus with tropism for skin, lymphocytes and sensory ganglia, where it establishes latency. Here, we identify large PML-NBs that sequester newly assembled nucleocapsids (NC) in neurons and satellite cells of human dorsal root ganglia (DRG) and skin cells infected with VZV *in vivo*. Quantitative immuno-electron microscopy revealed that these distinctive nuclear bodies consisted of PML fibers forming spherical cages that enclosed mature and immature VZV NCs. Of six PML isoforms, only PML IV promoted the sequestration of NCs. PML IV significantly inhibited viral infection and interacted with the ORF23 capsid surface protein, which was identified as a target for PML-mediated NC sequestration. The unique PML IV C-terminal domain was required for both capsid entrapment and antiviral activity. Similar large PML-NBs, termed clastosomes, sequester aberrant polyglutamine (polyQ) proteins, such as Huntingtin (Htt), in several neurodegenerative disorders. We found that PML IV cages co-sequester HttQ72 and ORF23 protein in VZV infected cells. Our data show that PML cages contribute to the intrinsic antiviral defense by sensing and entrapping VZV nucleocapsids, thereby preventing their nuclear egress and inhibiting formation of infectious virus particles. The efficient sequestration of virion capsids in PML cages appears to be the outcome of a basic cytoprotective function of this distinctive category of PML-NBs in sensing and safely containing nuclear aggregates of aberrant proteins.

## Author Summary

Many DNA viruses, including varicella-zoster virus (VZV), a herpesvirus that causes varicella (chickenpox) and zoster (shingles), replicate in the host cell nucleus. Here, we have identified an intrinsic antiviral mechanism that specifically targets newly assembled VZV capsids and contains these essential viral structures in a nuclear “safe house”. Using immuno-electron microscopy, PML (promyelocytic leukemia) protein fibers that formed filamentous spherical cages were shown to trap virion capsids very efficiently, preventing their transport out of the nucleus and inhibiting the formation of infectious virus particles. PML cages containing virion capsids were found in VZV-infected neurons and satellite cells in human sensory ganglia and in skin cells, which are major targets during VZV pathogenesis. Similar PML nuclear bodies that sequester abnormal proteins have been reported in neurodegenerative disorders, like Huntington's disease. We found that cages formed by PML isoform IV sequestered both the virion capsids of VZV, which is a neurotropic herpesvirus, and the mutant Huntington's disease protein. This work provides the first evidence that PML, which is abundant in mammalian cell nuclei, can function both to contain potentially damaging cellular protein aggregates and as an intrinsic host defense against a herpesvirus during nuclear virion assembly.

## Introduction

Promyelocytic leukemia protein (PML) is a major organizing component of structures that are referred to as PML nuclear bodies (PML-NBs) or nuclear domain 10 (ND10) bodies [1]–[3]. These nuclear bodies are heterogenous in size, shape and molecular composition [4]–[6], are prominent in most mammalian cell types and participate in many basic cellular functions, including transcriptional regulation [7], DNA repair [8] and apoptosis [9]–[12]. Human PML, located on chromosome 15, has nine exons and alternative splicing of PML transcripts produces at least 11 isoforms [13], [14]. PML isoforms share a conserved N-terminus, which has the characteristic RBCC/TRIM motif, including a RING finger domain, B boxes and a coiled coil domain. The PML N-terminus is important for PML heterodimer formation and oligomeriztion but each isoform has a unique C-terminal domain [14], [15]. The PML isoforms create PML-NBs with varying morphologies but the functional implications of these differences are not well understood [16]–[18]. While little is known about the tissue or cell specific patterns of expression of individual PML isoforms, these non-conserved PML regions are of interest for their likely involvement in isoform-dependent functions within particular cell types and in the response to changing intracellular or extracellular conditions [17].

Since their discovery, PML protein and PML-NBs have been investigated for their role in the virus-host cell interactions of DNA viruses that must replicate in the mammalian cell nucleus [19], [20]. These viruses include the very extensive Herpesviridae family as well as adenoviruses, papillomaviruses, polyomaviruses and other pathogens. During herpesvirus infection, genome copies are synthesized in nuclear replication compartments and genomic DNA is packaged into icosahedral nucleocapsids (NCs) formed by the major capsid protein and smaller capsid surface proteins. After assembly, NCs egress across the nuclear membrane for secondary envelopment in the cytoplasm and release as infectious virus particles [21], [22]. PML-NBs have been implicated in controlling the replication of herpes simplex virus (HSV) 1 and human cytomegalovirus shortly after virus entry by mechanisms that limit early viral gene transcription [19], [20], [23], [24]. To overcome these antiviral effects, HSV-1 targets PML for immediate proteosome-mediated degradation through functions of the viral ICP0 ubiquitin ligase protein [25]–[28]. PML is also an interferon (IFN)-inducible protein and must be degraded to prevent IFN-mediated inhibition of HSV-1 [29]. Like HSV-1, varicella-zoster virus (VZV) is a common human alphaherpesvirus [30]. In contrast to HSV-1 infection, PML protein and some PML-NBs persist in VZV-infected cells [31] although not in association with early nuclear replication compartments [32]. Depleting PML enhances VZV replication, suggesting a possible role for PML in the host cell defense [31].

VZV, which causes varicella (chickenpox) and herpes zoster (shingles), is a highly human-restricted pathogen [30], [33]. However, VZV pathogenesis can be investigated *in vivo* using xenografts of human dorsal root ganglia (DRG) and skin in a severe combined immunodeficiency (SCID) mouse model [34]–[36]. This model allows the analysis of viral and cellular mechanisms that facilitate or inhibit VZV when differentiated neural and skin cells are infected within their usual tissue microenvironments *in vivo* and in the absence of antiviral effects mediated by the adaptive immune response [37], [38]. The fundamental importance of intrinsic and innate cellular responses in achieving the usual balance between VZV and its human host is evident from the observations that VZV undergoes a transition to persistence in neurons within DRG xenografts without requiring VZV-specific adaptive immunity [36] and that VZV skin infection is highly regulated by type-I interferon produced by epidermal cells [39]. In these experiments, we have used the SCID mouse model to define VZV interactions with PML-NBs in human neural and epidermal cells infected with this nuclear replicating DNA virus *in vivo*.

The characteristics and functions of PML-NBs in human neural cells are of particular interest because VZV is a neurotropic herpesvirus [30]. Aberrant proteins generated in several neurodegenerative diseases, including Huntington's disease and spinocerebellar ataxias, accumulate in PML-NBs within neural cells and the usual nuclear distribution of PML is altered by their expression [40]–[45]. These diseases, classified as polyglutamine (polyQ) disorders, are associated with an unstable CAG repeat expansion resulting in the elongation of a polyglutamine (polyQ) tract in the abnormal gene product [46]. The mis-folded, oligomeric polyQ proteins are neurotoxic and form intranuclear aggregates, often with PML and proteins of the ubiquitin-proteasome system [47], [48]. Similar structures have been referred to as clastosomes [44], [49] and in some cases appear to protect the cell by confining the abnormal proteins to an intranuclear “safehouse” where they may be degraded [44], [50]–[52]. Here we report that endogenous PML forms distinctive, large PML-NBs consisting of spherical cages in the nuclei of neurons and satellite cells in human DRG and in skin cells during VZV pathogenesis. These nuclear organelles efficiently entrap newly assembled VZV capsids *in vivo* as well as in cultured cells *in vitro*. The PML-NBs closely resemble those that retain polyQ proteins in neurodegenerative diseases. Exogenous PML IV was the only isoform that sequestered VZV NCs and interacted with the ORF23 capsid surface protein, functions that required its unique C-terminus. PML IV-NBs also retained the Huntington's disease protein, Htt, and could simultaneously sequester NCs. Importantly, the nuclear entrapment of viral capsids in PML IV-NBs inhibited the production of infectious VZV progeny. These observations suggest that PML-NBs can retain nascent virion capsids in the infected cell nucleus, resulting in an intrinsic antiviral defense at later stages of viral replication.

## Results

### PML-NBs Colocalize with the ORF23 Capsid Protein in VZV-infected Cells

**Figure 1:**
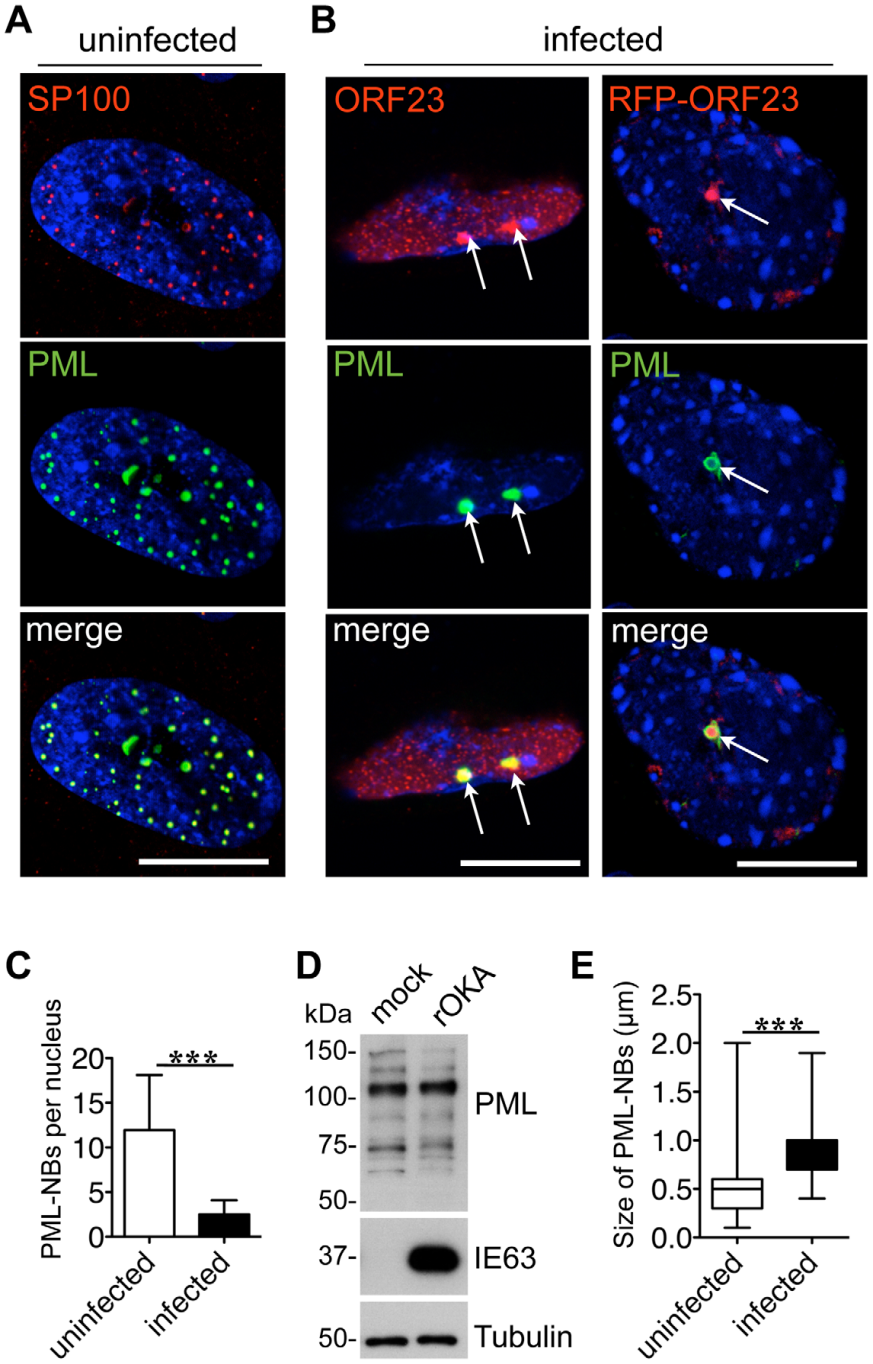
Endogenous PML-NBs colocalize with ORF23 capsid protein in VZV infected cells. (A) Uninfected human embryonic lung fibroblasts (HELF) were immunostained for SP100 (red) or PML (green) to visualize PML nuclear bodies (PML-NBs). Nuclei were stained with Hoechst (blue). (B) HELF cells were infected with cell-associated VZV for 24 hr and immunostained for PML (green) and ORF23 (red). PML-NBs and ORF23 capsid protein colocalized (white arrows) in HELF infected with either VZV (left panel) or VZV expressing red fluorescent protein (RFP)-tagged ORF23 (right panel). Nuclei were stained with Hoechst (blue). ORF23 capsid protein was identified in 94% (N = 228) of PML-NBs that were present in VZV infected cells at 24 hr. Scale bars in A and B are 5 µm. (C) Quantitation of the mean number of PML-NBs in nuclei of uninfected HELF (N = 180) and in infected HELF cells at 48 hr after infection (N = 180). The graph shows the mean ± SD; asterisks indicate a significant difference (p<0.0001). (D) Immunoblot analysis of PML protein (upper panel), VZV IE63 protein as a marker of VZV infection (middle panel) and tubulin (lower panel) in whole cell lysates of HELF that were mock-infected or infected with VZV (rOka) for 48 hr. (E) Quantitation of the mean size (diameter in µm) of PML-NBs in uninfected HELF and infected HELF. The box and whiskers plot shows the mean, 25 and 75 percentiles and the range of PML-NB sizes from 100 uninfected or infected nuclei, respectively; asterisks indicate a significant difference (p<0.0001).

Since PML protein and some PML-NBs persist in VZV infected cells [31], we first investigated the intracellular localization of VZV proteins that are found in nuclear viral replication compartments, including ORF29, the single stranded DNA binding protein and IE62, the major viral transactivating factor, as well as the ORF23 capsid protein, which is present in later stages of VZV infection when progeny virion assembly occurs [32]. As expected, analysis of uninfected human embryonic lung fibroblasts (HELF) by confocal microscopy showed numerous PML-NBs that contained both PML and SP100 protein, which are known components of PML-NBs [53](Figure 1A). In infected HELF, PML-NBs did not colocalize with viral DNA replication compartments, which were identified by ORF29 and IE62 expression [32], at this late time at 24 hr after infection (Figure S1A and B).

In marked contrast, the newly synthesized ORF23 capsid protein was present in almost all PML-NBs (94%, N = 228) at this stage of VZV infection (Figure 1B, left panel) using confocal imaging conditions that were optimized to identify bright PML-NBs associated with NCs. Based on its homology to the HSV-1 VP26 small capsid protein, ORF23 protein is predicted to be exposed on VZV NCs [54]. ORF23 protein was similarly enriched in PML-NBs in HELF infected with a VZV recombinant virus that expresses ORF23 protein tagged with the red fluorescent protein (RFP-ORF23) (Figure 1B, right panels) as well as in melanoma cells (Figure S2A). The number of PML-NBs decreased by about five-fold in both HELF and melanoma cells at 48 hr after infection compared to uninfected HELF and melanoma cells (Figure 1C and Figure S2B). Since melanoma cells have fewer PML-NBs than HELF before infection, the majority of infected melanoma cells had no PML-NBs left (Figure S2C). However, as previously reported [31], immunoblot analysis with a polyclonal anti-PML antibody showed that PML protein levels were not decreased in infected HELF or melanoma cells (Figures 1D and Figure S2D). The PML-specific bands were of varying sizes and may represent different PML isoforms or post-translational modifications of one or more of the PML isoforms. PML-NBs that colocalized with ORF23 protein in infected cells were also significantly larger than those in uninfected cells (Figure 1E). These results suggested that PML-NBs present at later stages of VZV infection sequestered ORF23 protein or possibly newly assembled NCs with surface ORF23 protein and might differ from the PML-NBs that modulate virus-cell interactions shortly after herpesvirus entry [55].

### VZV Nucleocapsids Are Sequestered in Endogenous Nuclear PML Cages

We next used cryoimmuno-electron microscopy (cryoimmuno-EM) to determine with ultrastructural precision whether the ORF23 protein that was associated with PML-NBs represented assembled NCs retained within these nuclear bodies or consisted of ORF23-PML protein co-aggregates. This question was of interest because PML has been observed to be associated with aberrant protein aggregates, termed nuclear aggresomes [56] or clastosomes [44] and with capsid proteins of other nuclear replicating viruses, including papillomavirus [57], [58], the neurotropic JC virus [59] and HSV-1 [60]. Except for the HSV-1 study, these analyses showed association of PML and capsid proteins by confocal light microscopy which does not optically resolve individual virion capsids; however some NC-like structures appeared to be associated with PML gold labeling in HSV-1 infected cells by immuno-EM [60].

**Figure 2:**
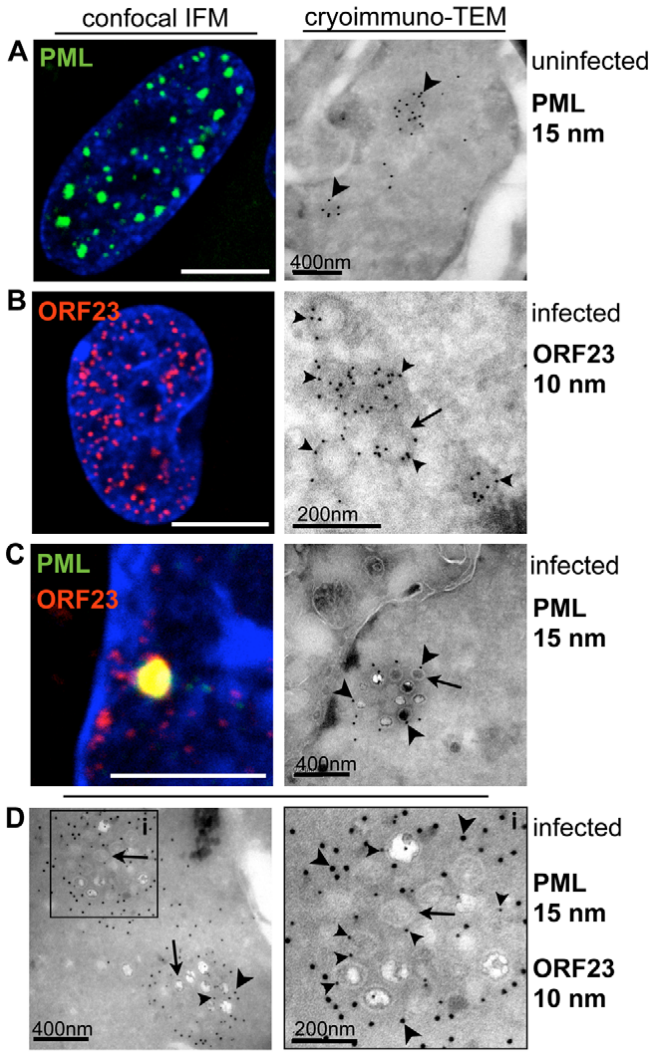
VZV nucleocapsids are sequestered in endogenous nuclear PML cages. HELF cells were uninfected (A, left panel) or infected (B, C, left panels) with VZV (rOka) for 48 hr and analyzed by confocal immunofluorescence (IF) microscopy after staining for PML (green) or ORF23 (red). For cryoimmuno-EM, HELF cells were uninfected (A, right panel) or infected (B–C, right panels; D, left and right panels) with VZV (rOka) for 48 hr and analyzed after immunogold labeling of PML (15 nm) or ORF23 (10 nm) by transmission electron microscopy (TEM). (A) PML-NBs visualized by confocal IF and by cryoimmuno-EM. Dense clusters of PML-specific gold particles (15 nm) label PML-NBs, indicated by arrowheads. (B) Tiny fluorescent punctae detected in VZV infected nuclei immunostained for ORF23 (red) by confocal IF may correspond to nucleocapsids (NCs). Cryoimmuno-EM identifies ORF23 (arrowheads) with NCs (arrows) by ORF23 specific gold-labeling of ring-like or hexagonal structures of about 100 nm diameter. (C) PML (green) and ORF23 (red) colocalize in PML-NBs shown by confocal IF microscopy. Cryoimmuno-EM in combination with PML-specific single-immunogold labeling (C, arrowheads) or double-immunogold labeling for PML and ORF23 (D, left panel) identifies clusters of VZV NCs (arrows) surrounded by PML protein. At a higher magnification (D, right panel) of the cluster indicated by the black square, NCs with ORF23-labeling (small arrowheads) and associated PML-labeling (large arrowheads) can be seen. Scale bars in IF images are 5 µm. Scale bars for EM images are as indicated.

Our experiments to detect both PML and ORF23 protein at ultrastructural resolution were first done with the Tokuyasu method which allows single and double-immunogold labeling of ultrathin (50–80 nm) cryosections with exquisite sensitivity [61]–[63]. These results were then correlated with patterns of PML and ORF23 protein localization observed by confocal microscopy (Figure 2). By cryoimmuno-EM, endogenous PML-NBs in uninfected cells were electron-dense subnuclear domains that exhibited extensive PML gold labeling, consistent with the punctate PML pattern seen by confocal microcopy (Figure 2A). In VZV infected cells, cryoimmuno-EM revealed VZV NCs of about 100 nm diameter (Figure 2B, arrow) that were abundantly labeled with antibody to ORF23 protein, confirming its predicted location on the capsid shell [54], [64], [65]. The viral NCs most likely corresponded to the tiny ORF23-expressing punctae seen in the nuclei of infected cells by confocal microcopy (Figure 2B, left panel). Furthermore, cryoimmuno-EM revealed clusters of VZV capsids that were surrounded by PML gold labeling (Figure 2C, right panel) and double-immunogold labeling proved the spatial association of PML protein (15 nm gold labeling) with individual NCs and clusters of VZV NCs detected with antibody to ORF23 protein (10 nm gold labeling) (Figure 2D). Therefore, the co-localization of PML-NBs and ORF23 protein detected by confocal microscopy (Figure 1 and Figure 2C, left panel) resulted from the sequestration of newly assembled VZV capsids in PML-NBs rather than from the formation of PML-ORF23 protein aggregates.

**Figure 3:**
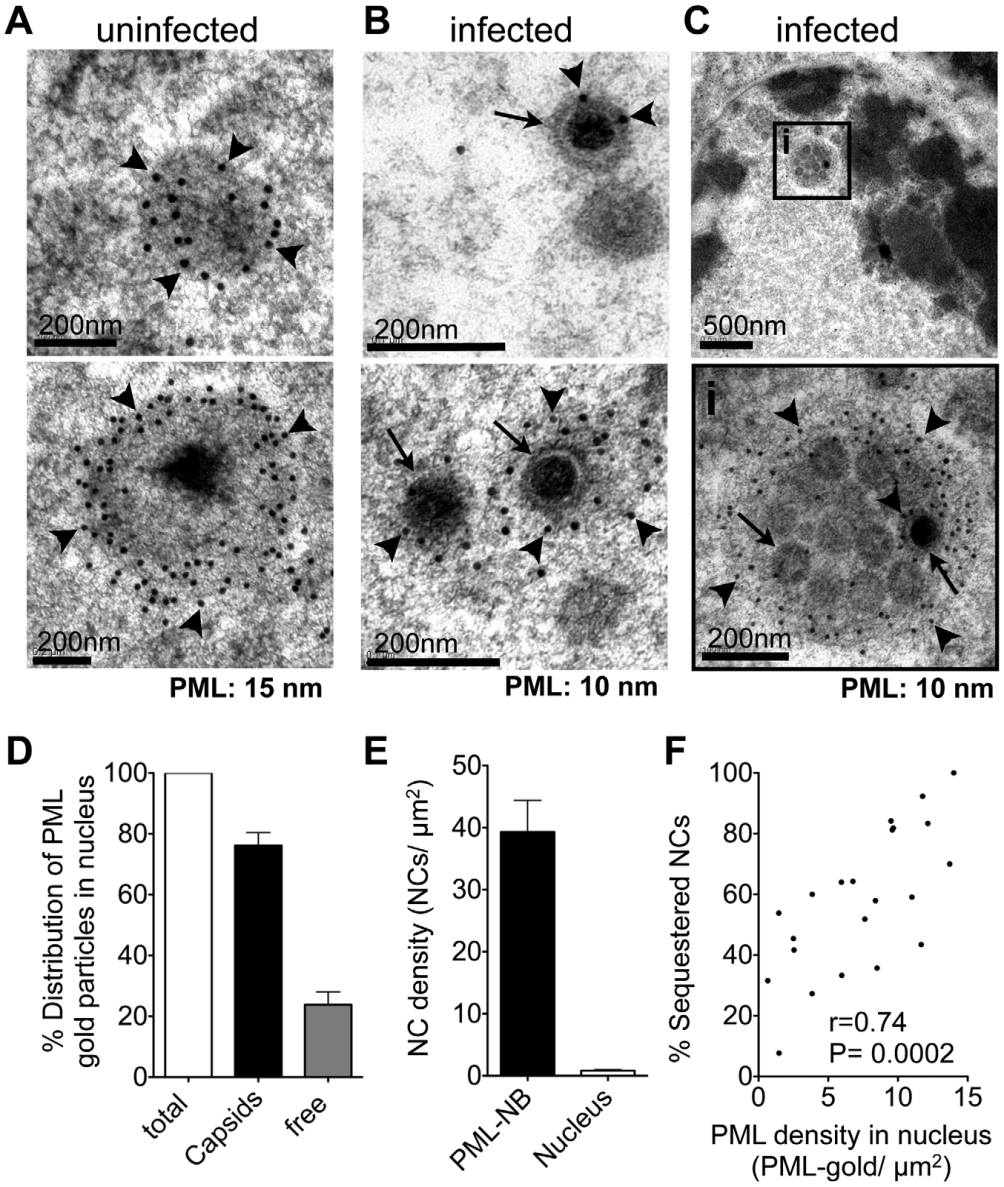
PML cages sequester mature and immature VZV nucleocapsids. (A-C) Representative immunogold-EM images of uninfected (A) or VZV-infected (B and C) HELF cells at 48 hr after infection and processing by high-pressure freezing (HPF) and freeze substitution (FS) to optimally preserve ultra-structural details. PML protein is specifically labeled with (A) 15 nm or (B and C) 10 nm gold particles (arrowheads indicate PML gold particles). Arrows indicate examples of viral NCs. (A) PML-labeled endogenous NBs in uninfected cells; examples are shown in upper and lower panels. (B) Individual VZV NCs (arrows) are associated with varying amounts of PML protein (arrowheads) as shown in the upper and lower panels. (C) Clusters of VZV NCs (arrows) are sequestered in an endogenous fibrous PML cage (arrowheads). The black square in the upper panel demarks the area (i) that is shown at higher magnification on the lower panel. Scale bars are as indicated. (D–F) Quantitative analysis of PML-specific gold particles (N = 1,611) and viral NCs (N = 450) in 30 VZV-infected cell nuclei. (D) Percentage of the total number of PML-specific gold particles associated with VZV capsids (black bar) or found ‘free’ within the nuclear area excluding PML-NBs (grey bar). (E) Density of viral NCs determined as NCs/µm^2^ (mean ± SD) within PML-NBs (black bar) or within the nuclear area outside PML-NBs (white bar). (F) Correlation between the percentage of NCs sequestered within PML-NBs (y-axis) and the PML labeling density in each nucleus, which is the number of PML-specific gold particles/µm^2^ of the total nuclear area (x-axis). Since only profiles of infected cell nuclei that contained at least 10 NCs were considered, N = 22 for this analysis. Pearson correlation: r = 0.74, R^2^ = 0.54, p<0.0002.

To define the ultrastructure of PML subnuclear domains harboring VZV NCs even more precisely, high-pressure freezing and freeze-substitution was used to optimally preserve both cell morphology and proteins [66] in uninfected and VZV infected HELF (Figure 3 and Figure S3). Again, endogenous PML-NBs in uninfected cell nuclei showed PML-specific labeling and were electron-dense (Figure 3A). In VZV infected cells, individual mature NC, which were identified by their dark electron-dense centers, as well as immature NCs, were associated with varying amounts of PML-positive fibrous material (Figure 3B and Figure S3). Furthermore, clusters of NCs were observed within cage-like structures that had dense PML-specific labeling (Figure 3C and Figure S3). These PML cages consisted of concentrically arranged protein fibers that tightly enclosed mature and immature NCs. Thus, the sequestration of virion capsids within PML subnuclear domains was confirmed with a second ultrastructural method. Furthermore, the presence of fibrous PML cages that entrapped both mature and immature herpesvirus capsids was demonstrated for the first time because of better contrast and superior ultrastructural preservation achieved with high pressure freezing and freeze-substitution.

Validating observations from EM studies requires quantitative analysis [67]. Examination of 30 VZV infected nuclei revealed that about 80% (N = 1,611) of all PML-specific gold particles were associated with viral NCs (Figure 3D). The concentration of NCs (number of NCs/µm^2^) within PML subnuclear domains was nearly 40 times higher than in other nuclear areas (Figure 3E), proving that PML and VZV capsids were highly co-enriched within PML-NBs and free PML protein was rare elsewhere in infected nuclei. In infected nuclei that contained endogenous PML-NBs an average of 62%±5 SEM (N = 30 nuclear profiles) of VZV NCs was sequestered in PML-NBs. The proportion of sequestered NCs (among all NCs present in an infected nucleus) was higher when more PML protein was assembled to PML cages and correlated positively with the amount of endogenous PML protein present in the infected cell nucleus, measured as the PML protein density (PML-gold/µm^2^) (Figure 3F). Importantly, the clusters of VZV NCs in these PML structures differed from the paracrystalline arrays of NCs that form nuclear inclusions in HSV-1 infected cells (compare Figure 3 and Figure S3 with Figure S4). Thus, the ultrastructural analysis unequivocally identified the association of both mature and immature virion capsids with PML protein in the nucleus and NCs were usually confined within endogenous cages consisting of PML-positive protein fibers in cells infected with VZV *in vitro*. Furthermore, these results demonstrated that immunogold EM is the method of choice to show the distribution of PML in infected nuclei unequivocally, including demonstrating the presence of PML on some individual NCs, that may be difficult to image by standard confocal microscopy. Immunogold EM has the significant advantage that individual gold particles constitute a stable, discrete and quantifiable signal that allows a very wide range of labeling densities within the same section to be imaged and quantified accurately.

### PML Cages Sequester Nucleocapsids During Infection of Human DRG and Skin Xenografts *In Vivo*

Since mature herpesvirus NCs must egress from the nucleus and undergo secondary envelopment in the cytoplasm before infectious virus release from the host cell [22], the sequestration of VZV NCs by endogenous PML cages suggested a novel mechanism by which PML might restrict viral replication and spread. However, this observation might reflect a phenomenon unique to cultured cells because VZV is known for its poor replication *in vitro.* Therefore, we next explored whether this cellular response also occurred in the differentiated neurons and satellite cells of sensory ganglia that are targeted during acute VZV infection *in vivo* [30] and when VZV reactivates from latency in neurons [68]–[70]. For these experiments, human DRG xenografts in SCID mice were infected for 14 days (acute phase of VZV infection [36]) and then harvested and processed for semithin (500 nm) cryosectioning and confocal microscopy or cryoimmuno-EM.

**Figure 4:**
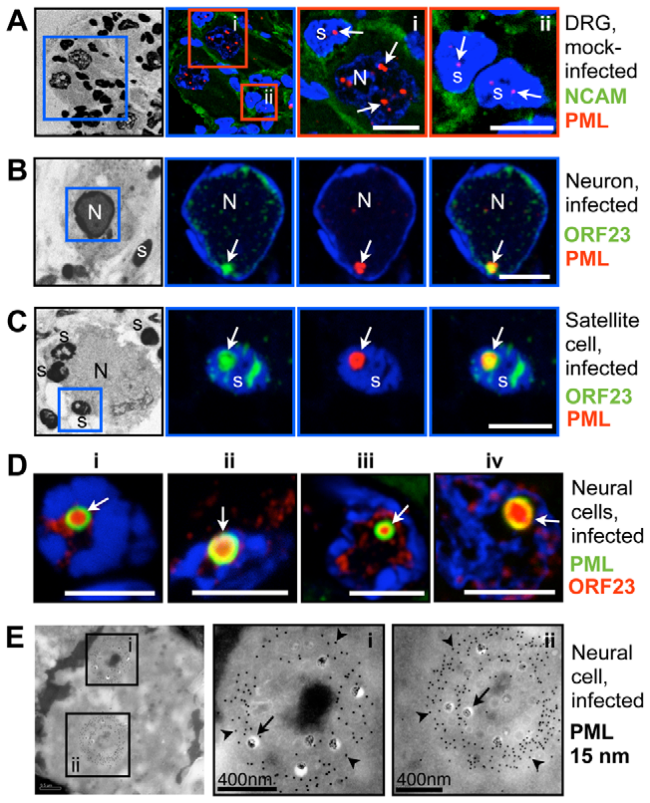
PML cages sequester VZV nucleocapsids during infection of human Dorsal Root Ganglia (DRG). Human DRG xenografts in the SCID mouse model were collected 14 days after mock infection (A) or inoculation with VZV (B–E) and fixed in 4% paraformaldehyde. Semithin (500 nm) cryosections were analyzed by confocal IF microscopy after staining for (A) Neural Cell Adhesion Molecule (NCAM) and PML, or (B–D) ORF23 and PML, as indicated at the right of each row of panels. Nuclei are stained with Hoechst (blue). Images in A-C (left panels) show overviews of the same DRG sections after Hoechst staining using an inverted grey scale. Blue or red squares demark areas shown at higher magnification in the adjacent panels. (A) Uninfected DRG sections show numerous small PML-NBs (red, white arrows) in the nuclei of neurons (N) and satellite cells (s). (B, C) VZV infected neurons and satellite cells with PML-NBs (white arrows) in which PML (red) colocalizes with ORF23 capsid protein (green). Scale bars are 5 µm. (D) Four examples (i–iv) of neural cells in infected DRG with large ring-like PML cages (green, white arrows) that sequester ORF23 protein (red). ORF23 capsid protein was present in 87% (N = 116) of PML-NBs within infected neural cells. Scale bars are 5 µm. (E) PML-specific immunogold-labeling of ultrathin (80 nm) cryosections of infected human DRG shows large ring-like PML cages (PML, 15 nm; arrowheads) that sequester numerous VZV NCs (arrows) in infected neural cells in human DRG. Areas in the black squares (i and ii) are shown at higher magnification in panels at the right. Sizes of scale bars are as indicated.

Numerous small PML-NBs (<1 µm) were detected in the nuclei of neurons and satellite cells in uninfected DRG by confocal microscopy (Figure 4A). In contrast, in infected neural cells, PML-NBs were either dispersed or reorganized such that only one or two enlarged structures were present; the mean diameter was (1.9 µm±0.8 SD; N = 62). These structures were often ring-shaped and exhibited intense enrichment of ORF23 protein in their interior (Figure 4B–D). ORF23 protein was present in a majority but not all (87%, N = 116) of the ring-shaped PML-NBs in VZV-infected neural cells. Ultrastructural studies revealed that these large PML-NBs consisted of spherical PML cages containing numerous sequestered viral NCs, as demonstrated by cryoimmuno-EM with PML-specific labeling (Figure 4E).

**Figure 5:**
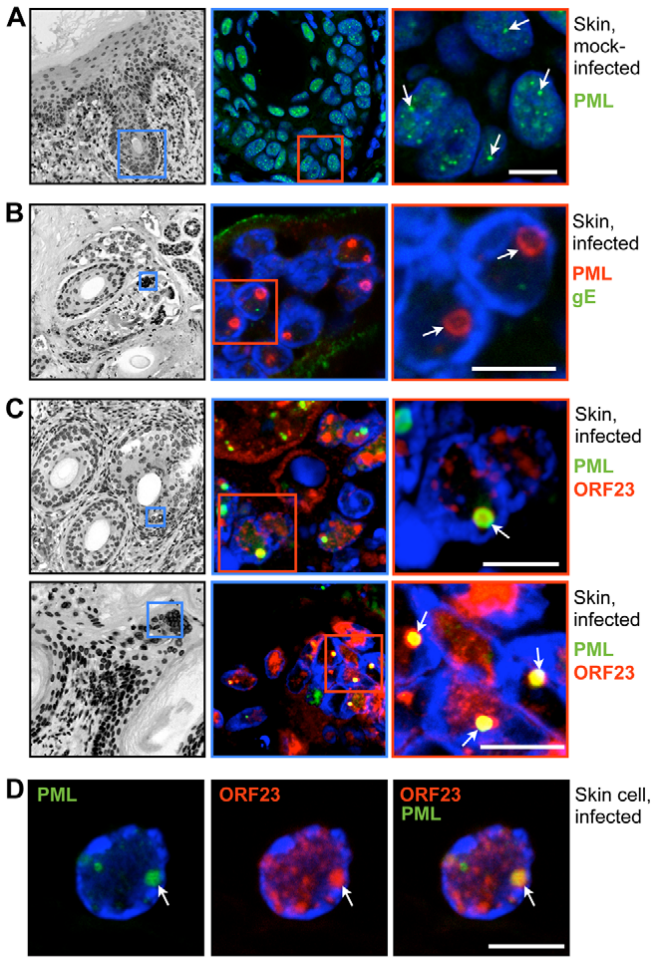
PML cages sequester VZV nucleocapsids during infection of human skin. Human skin xenografts in the SCID mouse model were collected 21 days after mock infection (A) or inoculation with VZV (B–D). Thick (5 µm) paraffin sections were analyzed by confocal IF microscopy after staining for PML (A), double-IF staining for PML and the VZV glycoprotein gE (B) or double-IF staining for ORF23 and PML (C and D), as indicated at the right of each row of panels. Nuclei are stained with Hoechst (blue). Images in A-C (left panels) are overviews of the same skin sections, including epidermal and dermal layers and hair follicles, after Hoechst staining (inverted grey scale). Blue and red squares demark areas shown at higher magnification in the panels on the right. Arrows indicate PML-NBs in the nuclei of skin cells. (A) Uninfected skin cell nuclei contain several small PML-NBs (green). (B) Infected cells have fused to form the syncytia (polykaryons). Large ring-like PML-NBs (red) are visible and VZV glycoprotein gE (green) is expressed on plasma membranes. (C) Large ring-like PML-NBs (green) sequester ORF23 protein (red). (D) A single skin cell nucleus showing PML-NBs (green) and ORF23 protein (red). All scale bars are 5 µm.

VZV also targets skin for replication, creating cutaneous lesions from which the virus is transmitted during varicella or herpes zoster [30]. To determine whether NC sequestration by PML-NBs also occurred in skin cells, skin xenografts were infected for 21 days, harvested and processed for paraffin-sectioning and confocal microscopy. Like uninfected neural cells, uninfected skin cells contained numerous small (<1 µm) PML-NBs (Figure 5A). However, very large ring-like PML-NBs were observed in the nuclei of infected cells that formed the typical VZV polykaryons and expressed the VZV glycoprotein gE on plasma membranes (Figure 5B); their mean diameter was 1.5 µm±0.3 SD (N = 81). Importantly, as observed in VZV infected human DRG cells, the ORF23 capsid protein was enriched in many of these large PML-NBs in infected skin cells (Figure 5C and D).

These experiments established that endogenous PML formed spherical nuclear cages containing VZV NCs not only in cultured cells *in vitro*, but also in differentiated human cells infected *in vivo*. Since neural and skin cells are targeted during VZV pathogenesis, we hypothesized that PML cages might play a role in the intrinsic host defense against VZV. These findings provided a rationale to further investigate the molecular mechanisms of this cellular response and to assess whether it could interfere with production of infectious virus progeny.

### Only the PML IV Isoform Promotes the Sequestration of VZV Capsids in Nuclear PML Cages

**Figure 6:**
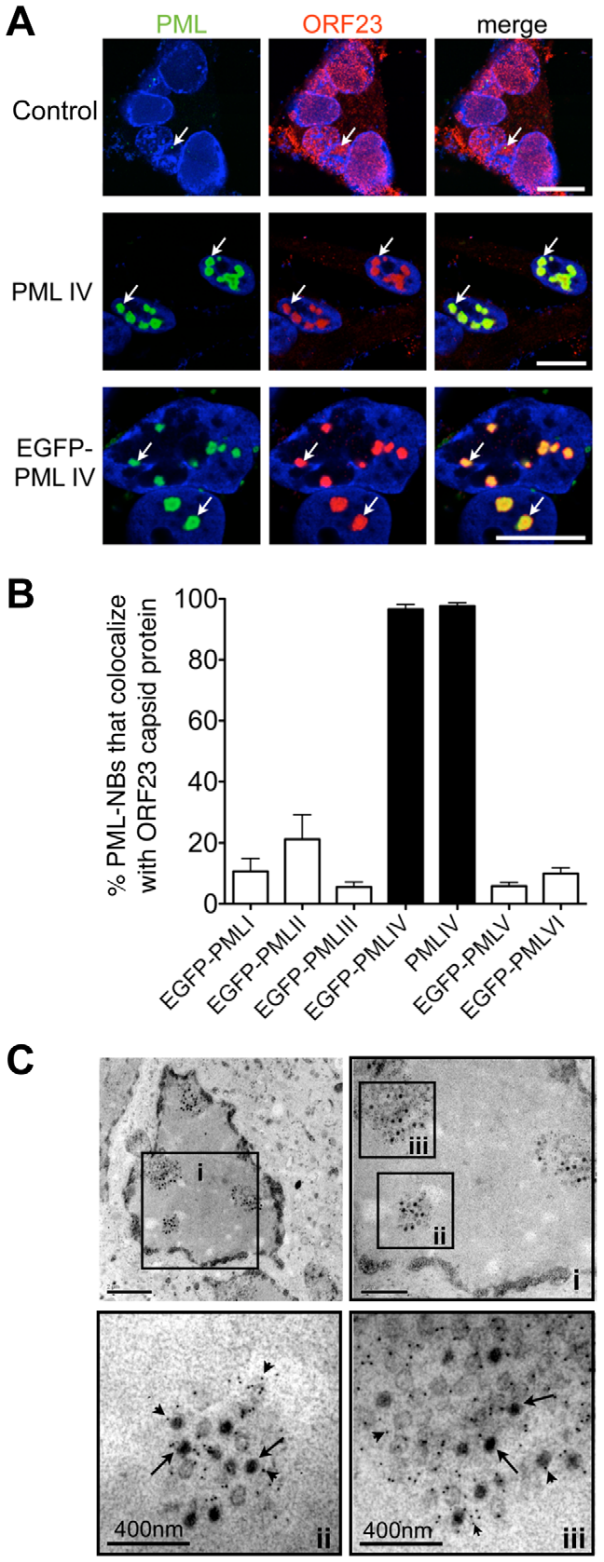
PML IV promotes the sequestration of VZV nucleocapsids within PML cages. (A) Representative IF-microscopy images show colocalization of PML IV-NBs (green) with ORF23 capsid protein (red) in VZV-infected melanoma cells at 48 hr after infection. Cells were either untransfected (control, upper panels) or transfected with plasmids expressing PML IV (middle panels) or EGFP-PML IV (lower panels). Nuclei were stained with Hoechst (blue). Arrows depict examples of PML IV bodies. (B) Quantitation of the percentage of PML-NBs that colocalize with ORF23 protein at 48 hr after infection of cells that had been transfected with plasmids expressing the six different PML isoforms, PML I-PML VI as indicated. Data shown for each PML isoform represent the mean percentage ± SD from five different samples with at least 100 PML-NBs each. (C) Representative immunogold-EM images after transfection with EGFP-PML IV and 48 hr after VZV infection. PML protein was identified with a polyclonal (rabbit) anti-PML antibody and Protein-A conjugated with 15 nm gold particles (small arrowheads). Three areas (i-iii, black squares) are shown at higher magnification. Arrows indicate viral NCs within densely labeled PML-NBs. Quantitative EM analysis of 100 infected nuclei showed that more than 90% of VZV NCs (N = 4,900) were sequestered in PML cages. EM scale bars are indicated.

PML protein exists in several isoforms that have unique C-terminal domains resulting from alternative splicing of the *PML* gene [14]. To define the process of NC sequestration in PML cages during VZV infection and to ask if this process might function as an intrinsic antiviral host defense, we evaluated six major PML isoforms, PML I, II, III, IV, V and VI. These experiments investigated whether exogenous expression of a particular PML isoform specifically altered the intranuclear distribution of ORF23 protein and sequestered VZV capsids. PML-NBs were formed by the exogenously expressed isoforms over the background of endogenous PML in uninfected and infected cells (Figure S5). In control cells that were not transfected but were infected with VZV, the ORF23 capsid protein showed no redistribution if endogenous PML-NBs were completely dispersed as happens in the majority of infected melanoma cells (Figure 6A, upper panel; see also Figure S2A). When melanoma cells were transfected with the PML isoforms and infected with VZV, ORF23 protein was redistributed and colocalized with more than 95% (N = 550) of PML-NBs only in cells that expressed PML IV or EGFP-PML IV (Figure 6A). None of the other five PML isoforms recruited ORF23 protein to PML-NBs (Figure 6B and Figure S5).

Photo-bleaching experiments with cells that expressed EGFP-tagged PML IV and that were infected with recombinant VZV expressing RFP-tagged ORF23 protein, revealed the striking immobilization of ORF23 protein within EGFP-PML IV-NBs. Even 45 min after photo-bleaching, RFP-ORF23 remained confined to PML IV-NBs (Figure S6), indicating that PML IV may form a physical barrier that constrains the mobility of ORF23 capsid protein and possibly of assembled capsids in the nucleoplasm. Therefore we next analyzed VZV infected cells expressing EGFP-tagged PML IV protein by immuno-EM to determine whether VZV capsids were confined in PML cages (Figure 6C). A quantitative analysis of 100 infected cell nuclei showed that more than 90% of VZV NCs (N = 4,900) were sequestered (Figure 6C) whereas other nuclear areas were essentially devoid of NCs, demonstrating a highly efficient retention of NCs in PML IV-NBs.

Furthermore, like endogenous PML cages, the exogenous PML IV cages (PML IV cages that formed in cells with endogenous PML and over-expressed PML IV) in VZV infected cells did not colocalize with the viral DNA replication compartments identified by IE62 and ORF29 protein expression or in situ hybridization for viral genomic DNA (Figure S7A–C). These experiments also demonstrated that ORF23 protein recruitment was specific, as compared to IE62 and ORF29 proteins, which showed no redistribution by PML IV.

### ORF23 Capsid Protein Interacts with PML IV in the Absence of Other Viral Proteins

Having shown that PML IV reorganized the nuclear distribution of ORF23 protein and sequestered NCs into large PML-NBs during VZV infection, we next investigated whether ORF23 protein was a molecular target of PML IV. ORF23 protein was considered a likely candidate for recognition by PML IV because, like the related HSV-1 VP26 small capsid protein [64], [65], ORF23 protein may decorate the capsid surface on hexons formed by the major capsid protein and is therefore likely to be accessible on NC surfaces. This prediction was supported by the dense ORF23-specific gold-labeling observed at the outer edges of VZV NCs (Figure 2B).

**Figure 7:**
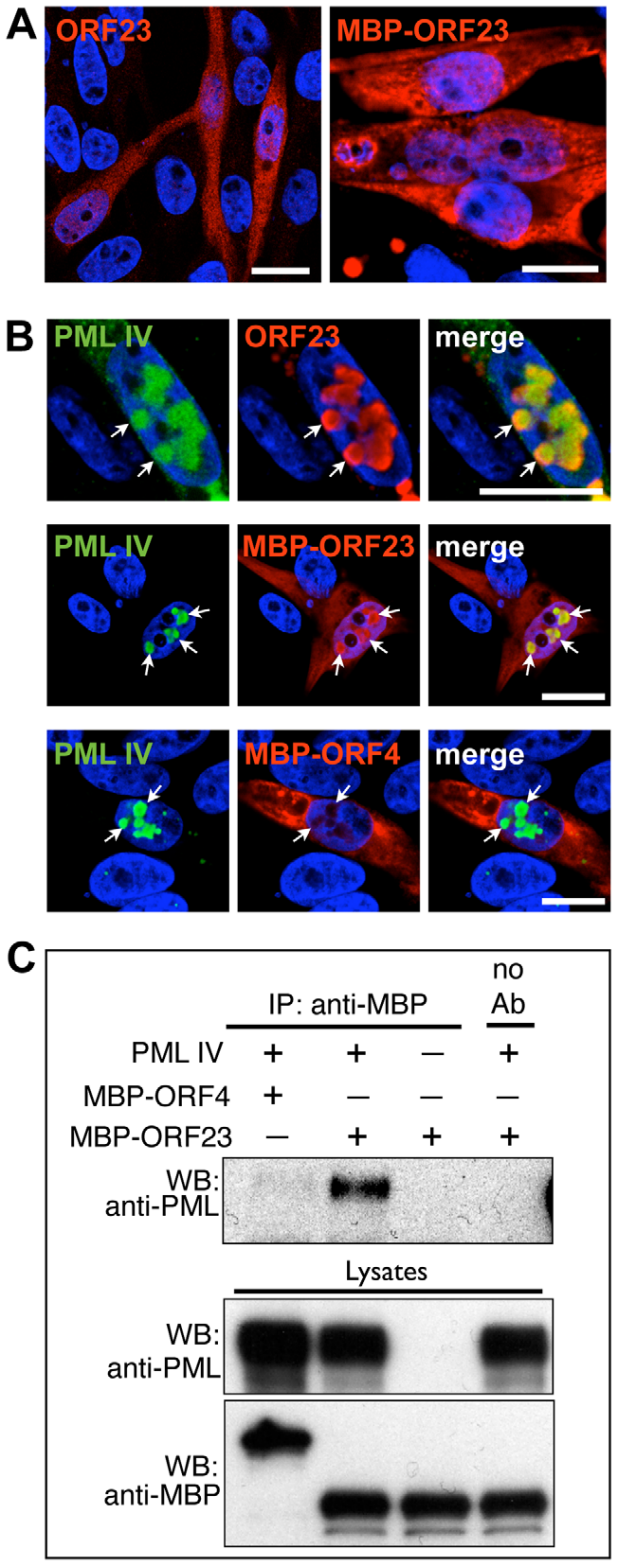
PML IV binds ORF23 capsid protein in the absence of other viral proteins. (A) Representative IF images show the cellular distribution of either untagged ORF23 (red; left panel) or maltose binding protein (MBP)-tagged ORF23 (red; right panel) at 48 hr after transfection of melanoma cells. (B) IF localization of untagged ORF23 protein (red; upper panels), MBP-tagged ORF23 protein (red; middle panels) or MBP-tagged ORF4 protein (red; lower panels) and PML IV bodies (green, white arrows) at 48 hr after transfection of melanoma cells. Nuclei were stained with Hoechst (blue). All scale bars are 5 µm. (C) Coimmunoprecipitation (Co-IP) of PML IV using an anti-MBP polyclonal (rabbit) antibody in cells co-transfected with PML IV expression plasmid and MBP-ORF23 or the control MBP-ORF4, in the combinations as indicated by ‘+’ and ‘-’. Co-IP of mock-transfected cells (no PML IV overexpression) is shown in the lane at the far right. PML IV was identified by immunoblotting with a polyclonal (rabbit) anti-PML antibody. Western blot of the lysates confirmed the expression of transfected PML IV, MBP-ORF23 and MBP-ORF4 (lower panel).

When expressed in melanoma cells, untagged ORF23 protein or ORF23 protein tagged with maltose-binding protein (MBP) protein was found to be distributed diffusely in both the cytoplasm and nucleus (Figure 7A). However, when cells were transfected with plasmids expressing PML IV, both ORF23 protein and MBP-tagged ORF23 protein were highly enriched within PML IV-NBs (Figure 7B). ORF4 protein, which is a VZV regulatory/tegument protein [71], [72], was used as an MBP-tagged control; it was not recognized by PML IV and did not colocalize with PML IV-NBs (Figure 7B, lower panels). The specific interaction of PML IV with MBP-ORF23 protein but not with MBP-ORF4 protein was confirmed by coimmunoprecipitation (Figure 7C). These data showed that ORF23 protein was recruited to PML IV-NBs in the absence of other VZV proteins. Therefore ORF23 protein is a potential molecular target on NC surfaces that may physically interact with PML IV during infection.

### The Unique C-Terminal Domain of PML IV Is Necessary for PML-mediated Sequestration of VZV Nucleocapsids

**Figure 8:**
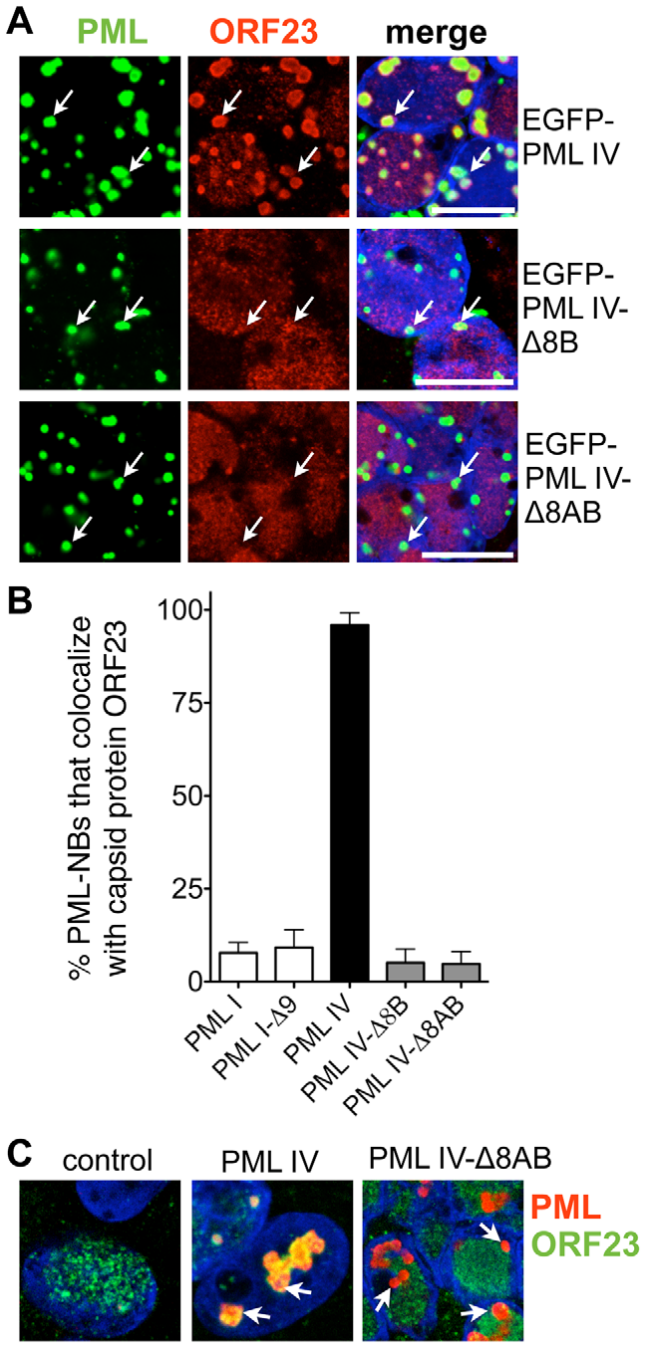
Sequestration of ORF23 capsid protein requires the C-terminal domain of PML IV. (A) Representative IF images show that EGFP-PML IV (green; upper panels) colocalized with ORF23 protein (red) but not with the C-terminal deletion mutants, EGFP-PML IV-Δ8B (green; middle panel) or EGFP-PML IV-Δ8AB (green; lower panel) in transfected cells that were infected with VZV for 48 hr. Nuclei were stained with Hoechst (blue). Arrows indicate the location of PML-NBs. Scale bars are 5 µm. (B) Quantitation of the percentages of PML-NBs that colocalized with ORF23 protein in infected cells that expressed the indicated PML isoforms or PML deletion mutants after transfection. The bars show PML-NB colocalization with ORF23 as the mean percentage ± SD for each PML isoform or deletion mutant from seven different areas, each of which contained 60-120 PML-NBs. (C) Doxycycline-inducible melanoma cells were generated that expressed no exogenous PML IV (left), PML IV (middle) or the truncated mutant PML IV-Δ8AB (right). The stable cell lines were induced with 5 µg/ml doxycycline and then infected with VZV for 24 hr. Cells were immunostained for PML (red) and ORF23 capsid protein (green); nuclei were stained with Hoechst (blue). White arrows indicate PML-NBs. Scale bar, 5 µm.

The unique C-terminal domain of PML IV, which is encoded by exons *8a* and *8b* of the *PML* gene, differentiates this isoform from the other five PML isoforms that failed to sequester NCs [14]. We therefore asked whether expression of truncated PML IV protein that had a deletion of exon *8b* (PML IV-Δ8B) or of both exons *8a* and *8b* (PML IV-Δ8AB) would eliminate the redistribution of ORF23 protein or capsids to PML cages. EGFP-tagged PML IV-Δ8B or PML IV-Δ8AB continued to form PML-NBs when exogenously expressed in VZV infected cells (Figure 8A). However ORF23 protein no longer colocalized with these mutant PML-NBs (Figure 8A and B), suggesting that the truncated PML IV proteins could not promote NC sequestration. PML I and the truncated PML I-Δ9 protein, lacking the unique PML I C-terminus encoded by exon *9*, were included as controls and also failed to cause redistribution of ORF23 protein (Figure 8B). We next investigated whether these results could be reproduced using stable melanoma cells lines that avoided EGFP-tagging and expressed untagged PML IV or PML IV-Δ8AB protein under the control of a doxycycline-inducible promoter (Figure 8C and Figure S8). These recombinant cell lines expressed similar amounts of PML IV and PML IV-Δ8AB protein at 24 hr after induction with 5 µg/ml doxycycline and formed PML-NBs that were similar in size (Figure S8A and B) and fewer than 0.5% cells exhibited apoptosis based on Annexin V staining (Figure S8C). When the induced cell lines were infected with VZV, ORF23 protein colocalized completely with PML IV at 24 hr after infection whereas ORF23 protein was not redistributed to PML-NBs in cells that expressed the truncated PML IV-Δ8AB (Figure 8C), again demonstrating that PML-NBs composed of truncated PML IV were deficient in the sequestration of ORF23 protein, presumed to be on VZV capsids.

**Figure 9:**
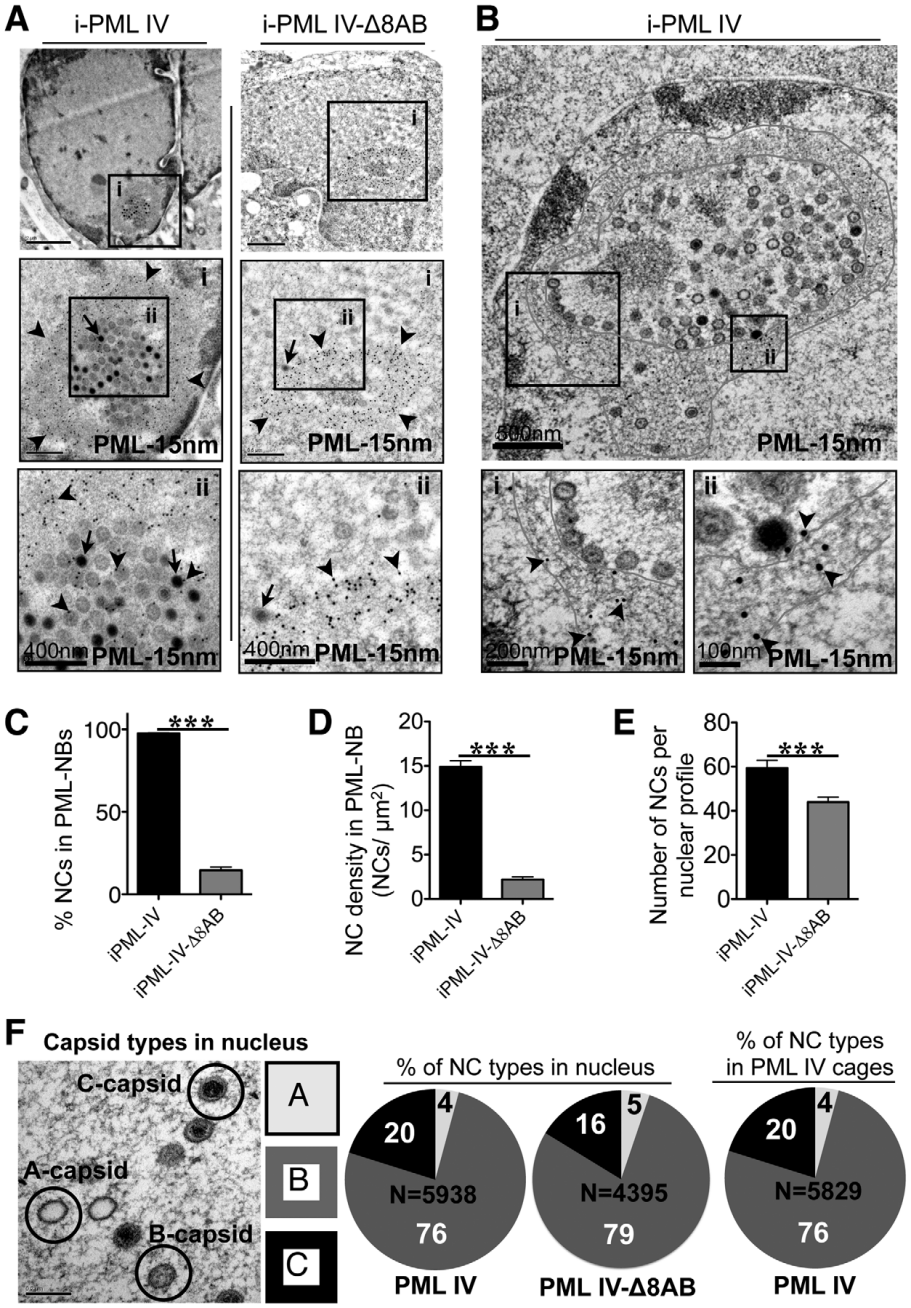
Quantitative ultrastructural analysis of PML IV cages with entrapped VZV nucleocapsids. (A) Cells induced to express PML IV (left panels) or PML IV-Δ8AB (right panels) and infected with VZV for 48 hr were processed for immunogold-EM. PML-NBs were identified with a polyclonal (rabbit) PML antibody and protein A conjugated to 15 nm gold particles. Areas in black squares (i and ii) are shown at higher magnification. Arrows indicate mature (C-type) capsids. Arrowheads indicate PML-specific gold particles (15 nm). The size of scale bars is indicated. (B) Cells induced to express PML IV and infected with VZV for 48 hr were embedded in Epoxy-resin. Ultrathin sections were surface-etched with hydrogen peroxide before PML-immunogold-labeling (15 nm, arrowheads). A PML IV cage with sequestered VZV NCs is shown. Red lines demark the outer and inner border of the spherical PML cage. PML-specific labeling (15 nm gold particles) is visible when the areas in the black squares (i and ii) are examined at higher magnification. The shell of the PML cage consists of PML-positive fibers (i and ii, lower panels). NCs align along the inner margin of the fibrous PML cage (area i). (C-E) Quantitative ultrastructural analysis of 100 nuclear profiles each in cells expressing PML IV (black bars) or PML IV-Δ8AB (grey bars). The total number of NCs analyzed was N = 5,938 in PML IV cells and N = 4,395 in PML IV-Δ8AB cells. (C) Quantitation of the number of VZV NCs shown as the percentage of the total NCs sequestered by induced PML IV (iPML-IV) and induced PML IV-Δ8AB (iPML IV-Δ8AB); the asterisks indicate a significant difference (p<0.0001). (D) Quantitation of the density of viral NCs within PML IV-NBs or PML IV-Δ8AB PML-NBs (mean ± SEM) within unit areas of PML-NBs (NCs/µm^2^) from an analysis of 100 PML-NBs (p<0.0001). (E) Comparison of the total number of VZV NCs per nuclear profile in nuclei with PML IV cages and nuclei with PML IV-Δ8AB NBs (mean ± SEM; p = 0.0003). (F) Proportion of the three types of VZV capsids within 100 nuclear profiles of cells induced to express PML IV or PML IV-Δ8AB and within PML IV cages. The left panel illustrates mature infectious (C-type), intermediate (B-type) and abortive (A-type) NCs visualized by TEM; the relative percentage of each type is shown in the pie charts (A-type: light gray; B-type: dark gray; C-type: black). The total number (N) of capsids analyzed is indicated in each pie chart.

To test this assumption, quantitative immuno-EM was used to show patterns of NC distribution in cells induced to express PML IV or PML IV-Δ8AB. For this purpose, samples were processed with high-pressure freezing and freeze-substitution to optimally preserve the ultrastructure of PML cages and virion capsids and to achieve sensitive detection of PML protein by immunogold labeling. Large spherical PML cages that harbored VZV NCs were readily observed in cells expressing PML IV (Figure 9A, left panel, and Figure 9B) and all PML IV cages entrapped both mature and immature NCs (Figure 9A, left panel; Figure 9B and Figure S9A–C). In contrast, PML IV-Δ8AB-NBs rarely sequestered any NCs even though numerous NCs were present in the infected cell nucleus (Figure 9A; right panel). Infected nuclei without any PML cages usually contained numerous randomly scattered NCs (Figure S9A; compare left and right panels).

The mean size of PML cages in infected cells induced to express PML IV was 1.6 µm ±0.5 SD (N = 100), which was similar to the sizes of PML cages in neural cells (1.9 µm ±0.8 SD; N = 62) and skin cells (1.5 µm ±0.3 SD; N = 81) in human tissue xenografts that were infected with VZV (Figures 4 and 5). However, PML IV-Δ8AB structures were significantly smaller than PML IV-NBs (1.2 µm ±0.4 SD vs. 1.6 µm ±0.5 SD; p<0.0001; N = 100) and had a more compact appearance than PML IV-NBs, presumably because fewer capsids were sequestered in these mutant PML-NBs. However, large PML IV cages that were only partly filled with sequestered NCs were also readily observed (Figure 9B) indicating that size and architecture of PML IV cages is not only determined by the sequestered cargo, but also by the assembly properties of PML-positive fibers itself. Thus, truncation of the unique C-terminal domain of PML IV influenced sequestration of NCs and the overall architecture of PML-NBs.

**Figure 10:**
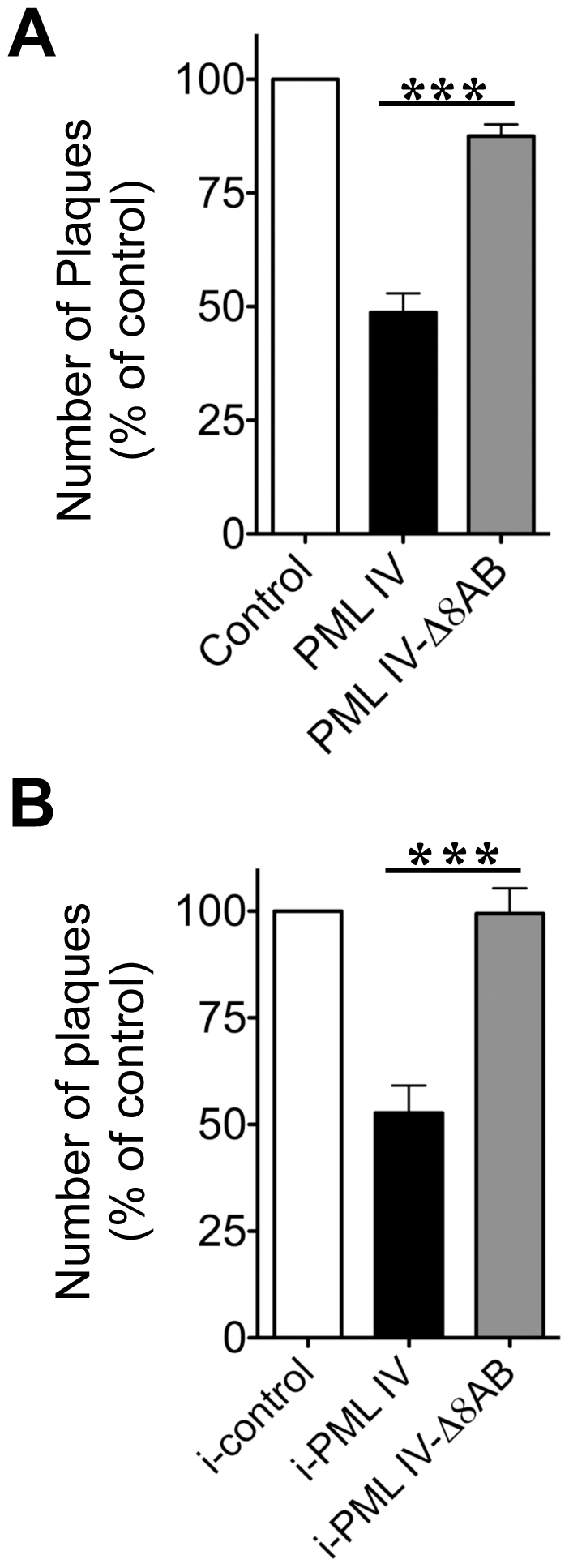
PML IV requires the unique C-terminal domain for inhibition VZV replication. (A) VZV plaque assay with melanoma cells that were transfected either with EGFP control, EGFP-PML IV or EGFP-PML IV-Δ8AB expression constructs and infected for 24 hr with VZV (rOka-RFP-ORF23) that expressed RFP tagged capsid protein. Only cells that were both transfected (green fluorescence) and infected (red fluorescence) were recovered by FACS. Melanoma cell monolayers were inoculated with equal numbers of cells from each of the sorted cell preparations. (A) Plaques were counted after 72 hr. Data are shown as the mean percentage (mean ± SEM) of plaque numbers normalized to the control (EGFP alone) from five independent experiments; plaque numbers were measured in triplicate in each experiment. The asterisks indicate a significant difference (p<0.0001). (B) Infectious virus production 24 hr after VZV infection of doxycycline-induced control, PML IV or PML IV-Δ8AB expressing cell lines, shown as mean plaque numbers (percentage of control ± SEM). The number of plaques formed in cells expressing PML IV was significantly reduced compared to the number in PML IV-Δ8AB expressing cells (p = 0.0018; N = 4).

Analysis of PML IV cages by TEM at high magnification revealed a shell structure consisting of PML-positive fibers that appeared to form a physical barrier entrapping VZV capsids (Figure 9B); NCs were often aligned along the inner side of the fibrous shell. PML IV cages were morphologically similar to the endogenous PML cages found in VZV infected HELF *in vitro* (Figure 2 and Figure 3) and in neural cells *in vivo* (Figure 4E). Importantly, the thin PML-positive fibers exhibited rather weak electron density (Figure 10B) and could only be identified unequivocally by high magnification and after PML-specific immunogold-labeling (Figure S9B and C). Therefore, despite their size, these PML structures would be easily be missed in virus-infected cells analyzed by morphological criteria only.

Quantitative analysis of NC distribution confirmed that PML IV cages in infected inducible cell lines were highly efficient in sequestration with more than 95% of NCs (N = 5,938) being retained in these structures (Figure 9C). In marked contrast, only 15% of NCs (N = 4,395) were found within PML-NBs in cell lines induced to express PML IV-Δ8AB (Figure 9C). The density of the packing of NCs within individual PML cages was also significantly higher in PML IV-expressing cells than in cells expressing PML IV-Δ8AB, suggesting that PML IV cages had more capacity to sequester NCs than those formed by truncated PML IV (Figure 9D). Furthermore, the total number of NCs was significantly higher (approximately 20%) in cells with PML IV cages compared to those with PML IV-Δ8AB-NBs (p<0.0001), indicating that NCs accumulated in PML IV-NBs (Figure 9E).

Herpesvirus capsids may be abortive (A type), intermediate (B type) or mature (C type) containing viral DNA genomes [73]. To determine whether NC assembly or packaging was effected by PML IV and if NC sequestration by PML IV cages was selective for a specific type of capsids, the relative proportion of the three major capsid types was quantified in nuclei expressing PML IV or PML IV-Δ8AB-NBs, as well as in PML IV cages (Figure 9F). Analysis of 5,938 NCs in 100 nuclei of cells expressing PML IV, of 4,395 NCs in 100 PML IV-Δ8AB expressing cells and of 5,829 NCs within PML IV cages, respectively, revealed similar proportions of all NC types; 16–20% of all NCs were mature, 76–79% were intermediate and 4–5% were abortive in each cell line and in PML cages (Figure 9F). This finding suggests that expression of PML IV did not increase the proportion of abortive or intermediate NCs compared to PML IV-Δ8AB expressing cells and that PML IV cages may participate in the antiviral host cell response against VZV by targeting all three types of NCs that are made in the infected cell nucleus for entrapment.

### PML IV Has Antiviral Activity against VZV that Requires the Unique PML IV C-Terminal Domain

Since PML IV restricted NCs to PML cages, we hypothesized that PML IV would impair the production of infectious virus. Conversely, we predicted that infectious virus yields would be unaffected in cells expressing the truncated PML IV-Δ8AB mutant, which does not recruit VZV capsids (Figures 8 and 9). Since VZV is highly cell-associated *in vitro* [74], viral replication is measured by plaque assays using infected cells as the inoculum. Given the critical importance of cell-cell spread for VZV infection, our objective was to assess the infectivity of VZV infected cells with and without PML-IV cages based on the transfer of virus from these cell populations to a permissive cell monolayer. We focused our analysis on the 24 hr time point after VZV infection of PML IV and PML IV mutant-expressing cells in order to avoid any potential effect of PML expression on the induction of apoptosis which could also reduce viral titers and because 24 hr is a late stage of VZV replication *in vitro* when NCs are present. We used two plaque assay methods to assess the antiviral activity of PML IV. In the first method, cells were transfected with an EGFP control, EGFP-tagged PML IV or PML IV-Δ8AB constructs, infected with recombinant VZV expressing RFP-tagged ORF23 protein for 24 hr and separated into subpopulations by fluorescent activated cell sorting (FACS). Only cells that emitted both green (EGFP, indicating transfection) and red (RFP, indicating infection) fluorescence were recovered and equal numbers of infected cells that expressed EGFP, EGFP-PML IV or EGFP-PML IV-Δ8AB were added to permissive cell monolayers and observed for plaque formation (Figure 10A–C). Plaques generated from inoculum cells that expressed PML IV were significantly reduced (p<0.0001) in five independent experiments; plaque numbers were determined in triplicate in each independent experiment. Plaque numbers were about 50% lower when compared to infected cells expressing PML IV-Δ8AB or the EGFP control (Figure 10A). Furthermore, the mean diameter of plaques that were created by infected cells expressing PML IV (0.95 mm^2^ ±0.06 SEM; N = 70) was significantly smaller than plaques produced by PML IV-Δ8AB expressing cells (1.46 mm^2^ ±0.09 SEM; N = 70) (p<0.0001). In contrast, the number and size of plaques from cells expressing PML IV-Δ8AB was very similar to the EGFP control (Figure 10A).

In the second method, the antiviral activity of PML IV was tested using the stable cell lines expressing inducible PML IV or PML IV-Δ8AB. These conditions avoided variables that might be associated with EGFP tagging of PML or infection with the recombinant virus in which ORF23 capsid protein was expressed as an RFP fusion protein. Inoculum cells that expressed induced PML IV and that were infected for 24 hr were added to permissive cell monolayers and observed for plaque formation (Figure 10B). Plaques generated from inoculum cells that expressed induced PML IV were significantly reduced (p<0.0018, N = 4) by about 50% when compared to infected cells expressing PML IV-Δ8AB or to control cells.

Thus, PML IV exhibited antiviral activity against VZV in transfected cells as well as in an inducible cell line and its antiviral activity was confirmed to require the unique PML IV C-terminal domain. The N-terminal region, which contains the RBCC/TRIM domain and is common to all PML isoforms and is present in the truncated PML IV mutant, was not sufficient for antiviral activity against VZV. Impaired virus production in PML IV expressing cells when compared to PML IV-Δ8AB and control cells correlated with the capacity of PML IV cages to sequester NCs as observed by immuno-EM. Conversely, the failure of the truncated PML IV-Δ8AB protein to exhibit antiviral activity correlated with impaired nuclear retention of NCs in VZV infected cells. These data together with the quantitative EM data that showed that more than 90% of NCs were present within PML cages at 48 hr suggest that NC are stably and not transiently contained in the PML IV cages.

### Individual PML IV Cages Co-sequester Capsid Protein and Huntington's Disease Protein in VZV Infected Cells

**Figure 11:**
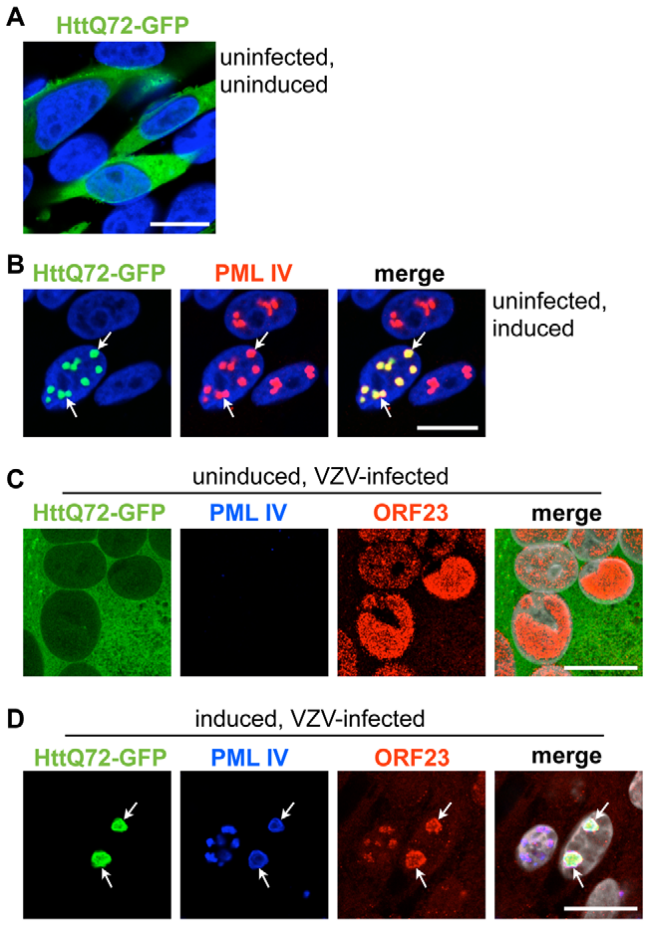
PML IV cages cosequester ORF23 capsid protein and Huntington's disease protein in VZV infected cells. PML IV inducible cell lines were transfected to express HttQ72-GFP. (A) HttQ72-GFP (green) expressing cells were fixed in the absence of PML IV induction. (B) HttQ72-GFP (green) expressing cells were induced to express PML IV and fixed after 48 hr. PML IV was detected by PML-specific IF staining (red). Recruitment of HttQ72-GFP to PML IV bodies (white arrows) is visible. (C) HttQ72-GFP transfected cells were infected with VZV for 48 hr without induction of PML IV expression. IF-images show the localization of HttQ72-GFP (green), PML (blue) and ORF23 capsid protein (red) in a syncytium (polykaryon) of fused VZV-infected cells. (D) A HttQ72-GFP transfected cell (the cell on the right) after induction of PML IV and infection with VZV for 48 hr. IF-images show the colocalization (white arrows) of HttQ72-GFP (green), PML (blue) and ORF23 capsid protein (red).

Ring-shaped PML-NBs similar to the endogenous PML cages in VZV infected neurons and satellite cells in human DRG and to PML IV cages *in vitro* have been observed in neural cell nuclei in neurodegenerative disorders associated with expanded CAG repeats encoding polyglutamine (polyQ) tracts in the abnormal gene product [41], [43]. Of particular interest, PML IV, which promotes VZV capsid sequestration has been reported to retain and degrade mutant polyQ-expanded Ataxin7 and the Huntington's disease protein (huntingtin or Htt) within ring-shaped PML clastosomes in cortical neurons [44]. We therefore asked whether the PML IV cages formed in our inducible melanoma cell lines were functionally like PML clastosomes as determined by their capacity to sequester GFP-tagged Htt [75]. We used the HttQ72 construct, which expresses the aberrant Htt protein with a 72 amino acid polyQ tract [75]. When PML IV cells were transfected without induction, HttQ72-GFP was primarily distributed diffusely in the cytoplasm (Figure 11A); it was also detectable in nuclei at higher levels of expression. This pattern changed dramatically when the cells were induced to make PML IV; almost all HttQ72-GFP was redistributed into nuclear PML IV-NBs (Figure 11B). This data confirmed observations about polyQ protein interactions with PML IV [44] and showed that PML IV-NBs in melanoma cells can sequester polyQ-tract proteins, as occurs in neuronal PML clastosomes.

These experiments led us to consider whether PML IV cages could co-sequester the ORF23 capsid protein marker, indicating retention of VZV capsids, together with the mutant Huntington's disease protein. VZV infection of cells that were transfected with HttQ72-GFP but not induced showed no change in the diffuse cytoplasmic pattern of HttQ72-GFP suggesting that VZV infection was not sufficient to redistribute this polyQ-protein (Figure 11C). However, when PML IV expression was induced, HttQ72-GFP was dramatically reorganized and colocalized in PML-NBs together with the ORF23 capsid protein in infected cells (Figure 11D). To our knowledge, these experiments demonstrated for the first time that the same PML isoform has the capacity to sequester both the aberrant poly-Q protein produced in a neurodegenerative disease and a viral capsid protein associated with NC entrapment in PML-NBs and that individual PML cages can simultaneously target and sequester an aberrant polyQ-protein and viral NCs.

## Discussion

Diverse types of PML-NBs that are distinguishable by their size, shape and dynamic behavior are found in mammalian cell nuclei [4], [5], [11], [18]. We identify a distinctive class of endogenous PML-NBs that form large spherical PML cages in differentiated human neural and skin cells infected with VZV *in vivo.* These endogenous PML cages were found to sequester newly assembled mature and immature viral nucleocapsids in the nuclei of infected neurons, satellite cells and epidermal cells, and in cultured cells infected with VZV *in vitro*.

Similar large PML cages that retained VZV NCs were formed when the PML IV isoform was expressed in cultured cells *in vitro* but not by PML isoforms I, II, III, V or VI. Importantly, PML IV exhibited antiviral activity against VZV. The antiviral function of PML IV was associated not only with a unique capacity to sequester NCs but also to bind the ORF23 capsid surface protein. All of these functions required the unique C-terminal domain of PML IV. The N-terminal region, which is common to all PML isoforms and contains the RBCC/TRIM domain [14] was not sufficient for antiviral activity or NC retention. The antiviral activity of PML IV was not likely to be explained by inhibition of viral genome synthesis or packaging into capsids by PML IV since the relative proportion of empty capsids was not increased in PML IV expressing cells. PML IV-Δ8AB may lack the capacity to produce PML-NBs with the architecture of those formed by intact PML IV, rendering them ineffective for capsid entrapment. It is also possible that the failure of PML-NB in PML IV-Δ8AB expressing cells to entrap virions reflects in part a dominant negative effect of PML IV-Δ8AB, preventing any endogenous PML IV from interacting with the ORF23 capsid protein.

The correlation between PML-mediated antiviral activity, capsid sequestration and ORF23 protein binding provides strong evidence that PML cages constitute an intrinsic host defense against this common herpesvirus. This role is supported by the finding that human neural and skin cells infected with VZV *in vivo* contained PML-NBs with the same distinctive morphology. ORF23 capsid protein was recruited to these structures and they consisted of cages formed by concentric rings of PML fibers sequestering NCs. Notably, while VZV infection promoted the dispersal of most PML-NBs, PML protein itself was not degraded, indicating that PML nuclear cages, rather than free PML protein, may be the primary obstacle to VZV replication.

How do NCs become entrapped within PML cages? Our working hypothesis is that individual NCs are recognized by PML protein (PML oligomers or fibers) through interactions with the capsid surface. These initial interactions may result in recruitment of more PML and NCs, thus growing into larger PML-NC assemblies. Alternatively, although they were rare in our immuno-EM studies, some aggregates of NCs may form spontaneously, which are then recognized by PML and enclosed in PML cages. Pre-existing, non-disrupted PML-NBs may also constitute a favorable environment (a platform) for NC aggregation, as has been proposed to account for the accumulation of aberrant cellular proteins in PML-NBs in neurons [45]. These possibilities are not mutually exclusive and a combination of all three may occur in the infected cell nucleus when capsids are produced.

Most PML cages observed in VZV infected neural and skin cells were significantly larger than the small PML-NBs that associate with viral genomes and exert antiviral effects by interfering with the initial transcription of herpesviral genes [76], [77]. PML cages were also more prominent later in infection, at the stage of viral capsid assembly. A significant antiviral effect was predicted because sequestration of abortive, immature and mature virions into fibrous PML cages was highly efficient, leaving very few mature capsids free to egress across the nuclear membrane to form infectious particles in the cytoplasm. Thus, PML cages conferred antiviral activity at a later stage of infection by a mechanism completely different from PML suppression of early viral gene transcription. These two PML-mediated antiviral mechanisms are not mutually exclusive and would be expected to function synergistically. The absence of PML cages may contribute to the increase in VZV replication observed when PML protein was depleted by RNA interference [31].

Our experiments provide the first evidence that a specific PML isoform, PML IV, contributes to the cellular antiviral response by promoting nuclear immobilization of herpesvirus nucleocapsids. PML IV interacted specifically with the ORF23 capsid protein in the absence of other VZV proteins. Since ORF23 protein was expressed on NC surfaces, it offers a likely target for PML IV recognition, as the first step leading to the retention of assembled NCs in PML cages. PML IV, as well as PML III and PML V has been reported to be expressed less abundantly than PML isoforms I/II in some cell lines and primary cells *in vitro* [17]. However, the pattern of PML isoform expression *in vivo* in human neural and skin cells for which VZV exhibits tropism or when these cells become infected with VZV is not known. We propose that PML IV may act as a potent modulator of PML-NB architecture, since others have also observed that exogenous PML IV (expressed along with endogenous PML) promotes the formation of large spherical PML-NBs [44]. As noted, PML cages that formed upon overexpression of PML IV together with endogenous PML *in vitro* best mimicked (both structurally and functionally) those endogenous large PML cages observed in neural and skin cells infected with VZV *in vivo* and both sequester NCs with ORF23 capsid protein. If PML IV is not abundant in the small PML-NBs of uninfected neurons, satellite cells or epidermal cells, the ratio of PML isoforms may change upon infection, PML IV may become more abundant or specific modifications on PML isoforms may be altered when these differentiated cells are infected or otherwise stressed. It was apparent that PML-NBs exhibited a striking reorganization from multiple small compact structures to a few large ring-shaped cages upon VZV infection in human tissues, which could be consistent with changes in the ratio of PML isoforms and upregulation of PML IV. Of particular interest, PML IV transcription was upregulated when cultured cells were stimulated with type I IFN [44], neurons have been shown to produce type I IFN during viral infection [78] and the IFN pathway is upregulated dramatically in human tissues infected with VZV *in vivo* [39]. Taken together, our findings point to the need to better understand the patterns and regulation of the tissue specific expression of PML isoforms and their functions in virus-infected cells. For example, different PML isoforms might recognize the outer capsid proteins of other herpesviruses or nuclear replicating DNA viruses and retain NCs, causing antiviral effects when a specific PML isoform is either not degraded efficiently or its production is induced in the infected cell.

Since the intranuclear mobility of herpesvirus NCs via actin filaments may facilitate formation of NC assembly domains and NC transport to the nuclear membrane [79], [80], we suggest that PML-mediated entrapment of NCs is likely to prevent NC interactions with the viral export machinery at the inner nuclear membrane. This hypothesis is supported by our observation that the number of intranuclear NCs was significantly higher in cells with functional PML IV cages as compared to control cells.

An intrinsic defense mechanism in which PML binds to a viral capsid protein and sequesters NCs obviously depends on the persistence or new synthesis of PML protein in the infected cell nucleus at the stage of capsid assembly, which occurs in VZV infected cells but not in HSV infected cells. Since HSV-1 degrades PML protein shortly after virus entry and shuts off host cell protein synthesis [25]–[27], both the early effects of PML on viral gene expression and this later antiviral activity would be expected to be counteracted. Some PML-specific gold labeling associated with NC-like structures, although not in PML cages, has been reported in HSV-1 infected cells at late times [60]. Overexpression of PML as a 69 kDa protein did not inhibit HSV-1 infection and PML cages were not observed but the PML isoform was not unequivocally identified [81]. Of interest, other nuclear replicating DNA viruses, including papillomavirus and JC (polymoma) virus also have limited capacity to degrade PML protein and PML binding to their capsid proteins has been reported [57], [59]. These interactions were proposed to support infection by directing the viral genome to replication compartments or facilitating virion assembly but enhancing or inhibitory effects of specific PML isoforms on viral replication could not be assessed [82], [83]. Nevertheless, particular PML isoforms could have pro-viral functions through some PML-capsid protein interactions and antiviral consequences in others, as we observed here in VZV infected cells.

Interestingly, stimulation of PML expression by type-I interferon and PML-mediated sequestration and immobilization NCs of VZV, a DNA virus, has parallels with the antiviral mechanism of IFN-induced Mx-GTPases against RNA viruses. These proteins also form oligomeric structures and inhibit the replication and assembly of several negative-strand RNA viruses by sequestering and immobilizing viral ribonucleocapsids or nucleocapsid protein [84]. For example, the human MxA GTPase was found to sequester the LaCrosse bunyavirus nucleocapsid protein into fibrillar cytoplasmic complexes thereby inhibiting viral RNA genome replication and the formation of enveloped virion particles on membranes in the Golgi compartment [85], [86]. Thus, these two IFN-induced antiviral responses appear to depend on creating intracellular structures that act as physical barriers during the later viral assembly stage of infection.

Our observations of VZV NC sequestration in PML-NBs in neurons, satellite cells and skin cells *in vivo* are relevant to a better understanding of the control of VZV infection in the human host. We suggest that the role of NC entrapment by PML cages in skin is important for the modulation of the infection by the innate, interferon-induced response through restriction of cell-cell spread. Diverting a substantial majority of mature capsids to PML cages is likely to regulate lytic VZV infection *in vivo*. In fact, this process may benefit the virus since it is important for VZV and other herpesviruses to achieve a well-balanced virus-host interaction. Persistence of the virus in the human population requires a relatively mild infection. An incapacitating infection of the host that would limit opportunities for VZV transmission to susceptible contacts and would hinder, rather than support the persistence of the virus in the population as a whole. In addition to limiting skin infection, the consistent finding of large PML cages in neuronal cells of human DRG xenografts infected with VZV suggests the possibility that this intrinsic host cell response might restrict episodes of VZV reactivation from latency so that the episode remains subclinical [33], [87]. We hypothesize that when VZV reactivates from latency in persistently infected neurons, trapping newly formed NCs by PML cages would reduce the possibility for spread of infectious virus particles down the axons to skin and would also limit infection of adjacent satellite cells and neurons in the DRG where reactivation is occurring. This process would result in abortive VZV reactivation [88]. From a clinical perspective, it is notable that patients treated with arsenicals have an extremely high rate of clinically symptomatic herpes zoster, due to VZV reactivation [89], [90] and arsenicals are potent triggers of PML degradation [91], [92].

Since VZV is a human neurotropic virus, it is of interest that ring-like PML-NBs similar in size to endogenous PML cages in VZV infected neurons and satellite cells in DRG *in vivo* and PML IV cages *in vitro* are found in neural cell nuclei in several neurodegenerative disorders [45]. Many of these disorders are associated with expanded CAG repeats encoding polyglutamine (polyQ) tracts, including Huntington's disease and several types of spinocerebellar ataxia and PML was associated with these protein inclusions [40]–[43]. Importantly, the same PML isoform, PML IV, which promotes VZV capsid sequestration has been reported by others to form ring-like PML clastosomes that mediate the sequestration and degradation of mutant polyQ-expanded Ataxin7 and Huntington's disease protein (huntingtin or Htt) in cortical neurons [44]. The morphological similarity between PML cages found in VZV infected cells and these ring-like PML clastosomes and the finding that PML IV can co-sequester ORF23 capsid protein together with the Huntington's disease protein in VZV infected cells, suggest that the PML cages described here and PML clastosomes are structurally and functionally related subnuclear domains. Neurons are post-mitotic cells and need to safely contain toxic proteins and/or increase their clearance, whether aberrant host cell proteins or virion structures [46], [50]. Of interest, aberrant polyQ proteins tend to form large aggregates that like NCs may be particularly resistant to the celluar mechanisms for degradation of unwanted proteins and the nucleus may have limited capacity to cope with misfolded proteins compared to the cytoplasm [75]. PML IV may specialize in restricting these degradation-resistant structures to nuclear subdomains and limit their toxicity longer term.

In summary, the efficient sequestration of virion capsids in PML cages that contributes to the intrinsic antiviral host response against VZV appears to be the outcome of a basic cytoprotective function of this distinctive category of PML-NBs in safely containing aggregation-prone aberrant proteins. Both aggregation-prone proteins and virion capsids are therefore potential substrates for sequestration in these PML cages.

## Materials and Methods

### Ethics Statement

Human fetal tissues for SCID xenograft studies were obtained from Advanced Bioscience Resources, Inc. (Alameda, CA) in compliance with state and federal regulations for tissue acquisition for biomedical research, in accordance with FDA 21 CFR Part 1271 GTP (Good Tissue Practices), UAGA and NOTA. Animal protocols complied with the Animal Welfare Act and were approved by the Stanford University Administrative Panel on Laboratory Animal Care.

### Cells and Viruses

Human lung embryonic fibroblasts (HELF) and a human melanoma cell line (MeWo, ATCC number: HTB-65) were grown in Dulbecco's modified Eagle's medium supplemented with 10% fetal bovine serum, nonessential amino acids (100 µM) and antibiotics (penicillin at 100 U/ml and streptomycin at 100 µg/ml). HELF and the melanoma cell line (as obtained from ATCC) were passaged less than 25 times.

VZV used in these experiments was recombinant Oka (rOka) derived from the wild type low passage parent Oka strain (pOka). VZV (rOka) and the recombinant rOka-RFP-ORF23 expressing RFP-tagged ORF23 [54] were propagated in melanoma cells and HELF cells [32]. Transfection was done with Lipofectamin 2000 (Invitrogen) for 14 hr. Viral infection was done with cell-associated VZV at a ratio of 1/20 (infected cells/uninfected cells) for 24 or 48 hr.

### Expression Plasmids

Plasmids pEGFP-PML (I-VI) [18] were gifts from Prof. Peter Hemmerich, Leibnitz-Institute of Age Research, Jena, Germany**.** Plasmid pcDNA3-PML IV [44] was generously provided by Prof. Annie Sittler, Universite Pierre et Marie Curie, Paris, France. Plasmids pCR3-MBP-ORF4**,** pCR3-MBP-ORF23 and pENTR207-ORF23 vectors have been described [93]. The plasmid pcDNA3-HttQ72-GFP [75] was a kind gift from Prof. Ron Kopito, Stanford University, Stanford, USA. Plasmid pCR3-ORF23 that expresses untagged ORF23 was constructed using the pENTR207-entry vector and the pCR3 destination vector with the LR Clonase II gateway cloning reaction (Invitrogen). PML deletion constructs EGFP-PML I-Δ9, EGFP-PML IV-Δ8AB and EGFP-PML IV-Δ8B were made as follows: exon 9 was removed from PML I by PCR (primers PML IΔ9 5′A/PML IΔ9 5′B and PML IΔ9 3′A/PML IΔ9 3′B). pEGFP-CI PML IV was the template used to delete exon 8b (primers PML IVΔ8B 5′A/PML IΔ9 5′B and PML IV 3′A/PML IV 3′B) and exons 8ab (primers PML IVΔ8AB 5′A/PML IVΔ8AB 5′B and PML IV 3′A/PML IV 3′B). pEGFP-C1 PML I was digested with MluI and a 6.1 kb vector DNA was isolated. pEGFP-C1 PML IV was digested with BbvCI/MluI and a 5.6 kb vector DNA was isolated. Purified, digested PCR products were ligated into the appropriate vectors to generate pEGFP-PML I-Δ9, pEGFP-PML IV-Δ8AB and pEGFP-PML IV-Δ8B. Primer sequences are listed in Table S1.

### Construction of Inducible PML Expressing Melanoma Cell Lines

Melanoma cells expressing doxycyline-inducible PML IV or PML IV-Δ8AB were made using the pRetro-X-Tet-On-Advanced vector system and pRetro-X-Tight-Pur plasmid (Clontech Laboratories) with the PML IV or PML IV-Δ8AB sequence derived from the plasmid pcDNA3-PML IV [44]. The stable cells were induced with 5 µg/ml doxycyline for 24 hr before infection with VZV.

### Human Dorsal Root Ganglion (DRG) and Skin Cell Xenografts

Human fetal DRG were inserted under the kidney capsule of male CB-17^scid/scid^ mice (Taconic Farms, Germantown, NY), inoculated with rOka-infected HELF and harvested after 14 days [36]. Human fetal skin xenografts were inoculated with VZV rOka-infected HELF and harvested after 21 days [34]. Human tissues were provided by Advanced Bioscience Resources (ABR, Alameda, CA) and were obtained in accordance with state and federal regulations. Animal protocols complied with the Animal Welfare Act and were approved by the Stanford University Administrative Panel on Laboratory Animal Care.

### Microscopy Analysis of Xenograft Tissue Sections and Cultured Cells

DRG xenografts were prepared for cryosectioning as described previously [88]. Human skin xenografts were embedded in paraffin and 5 µm sections were prepared. For confocal microscopy, sections were deparaffinized with tissue clearing agent (“Safeclear II”, Fisher Scientific), rehydrated and antigen retrieval was performed in citric acid-based antigen retrieval solution for 90 sec at 100°C. Sections were then blocked and immunolabeled as described previously [88]. Cultured cells on glass coverslips were fixed in 4% paraformaldehyde in PBS for 20 min at room temperature or were fixed with methanol at -20°C for 10 min and air-dried. Cells were blocked and immunostained as described previously [32].

### Antibodies and Confocal Immunofluorescence Microscopy

Antibodies used for confocal microscopy were: rabbit polyclonal anti-PML and mouse monoclonal anti-PML (PG-M3), rabbit polyclonal anti-Sp100 (all Santa Cruz Biotech), mouse monoclonal anti-N-CAM (Zymed), rabbit polyclonal anti-VZV-ORF23 [54], rabbit polyclonal anti-VZV-IE62 and rabbit polyclonal anti-VZV-ORF29 [32]. Antibodies for secondary detection were Alexa Fluor 488, Alexa Fluor 594 or Alexa Fluor 647 conjugated donkey anti-mouse or donkey anti-rabbit antibodies (Invitrogen). Infected tissues, cultured cells and PML-NBs were imaged and quantified using a Leica TCS^SP2^ confocal laser scanning microscope (Heidelberg, Germany). Microscope objectives were 40/1.0 (Numerical Aperture, N.A.) or a 63/1.4 (N.A.) plan apochromate objectives. Images were scanned at 1024×1024 pixels with at least four times frame averaging and the pinhole adjusted to one airy unit. Brightness and contrast were adjusted using Photoshop CS3 (Adobe) or iPhoto (Apple). The number of PML-NBs and colocalization of PML-NBs with ORF23 protein were quantified using captured digital images and the analysis and measurement tools in Photoshop CS3 extended version (Adobe).

### Electron Microscopy (EM)

Samples were either prepared for cryosectioning or high-pressure frozen (HPF) and freeze substituted (FS) and then embedded in either LR-white or Epoxy resin (Embed812). Preparation and labeling of cryosections was done as described previously [88]. Cells were high-pressure frozen in a Leica EM PACT2. Frozen specimen carriers with cells were placed into frozen cryovials containing acetone with 0.1% glutaraldehyde and 0.1% uranyl acetate (for LR White embedding) or in acetone with 1% osmium tetroxide and 0.1% uranyl acetate (for Epon embedding). The frozen vials were placed into a Leica AFS for the freeze-substitution procedure and then embedded in either epoxy resin (Embed 812) or LR-White resin. Ultrathin (50–70 nm) LR-white or Epon-sections were prepared with a diamond knife (Diatome) using a ultramicrotome (Ultracut, Leica). For immunogold-labeling of Epon-sections, these sections were first etched with 5% hydrogen peroxide for 5 min and then rinsed with distilled water. Sections were pre-blocked in DIG-blocking solution (Roche) for 30 min. Primary antibodies and Protein A-gold particles were diluted in blocking solution and sections were incubated for 1 hr or 30 min, respectively, at RT. Rabbit polyclonal anti-PML antibody (Santa Cruz Biotech) and rabbit polyclonal anti-ORF23 antibody were used at 1∶10 dilution. Finally the sections were stained with 3.5% aqueous uranylacetate for five minutes and with 0.2% lead citrate for three minutes and air-dried. Sections were analyzed using a JEOL 1230 transmission electron microscope (TEM) at 80 kV and digital photographs were taken with a GATAN Multiscan 701 digital camera. Quantification of PML-specific immunogold-labeling and evaluation and counting of virion capsids was done by analyzing captured digital images with Photoshop CS3 (extended edition, Adobe) using the counting tool and the measuring tool to calculate areas of nuclear profiles and PML compartments. PML-labeling density (number of gold particles per µm^2^) and capsid density (number of capsids per µm^2^) were calculated with Microsoft Excel (2008). Statistical analysis was performed using GraphPad Prism (version 5.0) statistical software.

### VZV Plaque Assays

For plaque assays after fluorescent activated cell sorting (FACS), cells were transfected with pEGFP, pEGFP-PML IV or pEGFP-PML IV-Δ8AB expression plasmids, infected for 24 hr with rOka-RFP-ORF23 and sorted by FACS (Digital Vantage, BD Bioscience). Cells that were both green (transfected) and red (infected) were recovered, equal numbers were seeded onto melanoma cell monolayers in triplicate and plates were incubated for 72 hr. Plaques were visualized by standard methods; five independent FACS experiments were performed. Plaque numbers were counted and plaque sizes were determined from digital photographs by calculating the area of >70 randomly selected plaques per sample using Photoshop CS3 (extended edition, Adobe). Virus plaque assays with inducible PML expressing cell lines (Mel39-control, Mel39-PML IV and Mel39-PML IV-Δ8ab) were done using cells induced overnight with 5 µg/ml doxycycline, infected for 24 hr, harvested, serial diluted and seeded in triplicate onto melanoma cell monolayers. Plaques were counted and numbers expressed as percentages of the control; four independent experiments were performed.

### Immunoprecipitation (IP) and Western Blotting

For immunoprecipitation studies, samples were analyzed using the ExactaCruz IP-Western Kit (Santa Cruz Biotechnology, Inc.), according to manufacturer's instructions. MBP-ORF23 or MBP-ORF4 and PML IV transfected cells were grown in 75 cm^2^ flasks and lysed in 300 µl high salt buffer (20 mM HEPES [pH 7.2], 450 mM NaCl, 1.5 mM MgCl_2_, 0.5% NP-40, 20% Glycerol) supplemented with EDTA-free protease inhibitor cocktail (Roche). Cell debris was removed by centrifugation. The supernatant was combined with 1,200 µl NaCl-free low salt buffer (20 mM HEPES [pH 7.2], 1.5 mM MgCl_2_, 0.5% NP-40, 20% Glycerol) and centrifuged again. 4 µg of anti-MBP monoclonal antibody (New England Biolabs) was coupled to 100 µl sepharose beads. Beads were washed in PBS and then incubated with 1,000 µl pre-cleared cell lysate at 4° C for 4 hr. Beads were then washed six times with the binding buffer (20 mM HEPES [pH 7.2], 90 mM NaCl, 1.5 mM MgCl2, 0.5% NP-40, 20% glycerol), boiled with SDS sample buffer, and analyzed by Western blot using rabbit polyclonal anti-PML antibody (1∶2,000), anti-MBP antibody (1∶2,000). For Western Blot analysis of cultured cell lysates, cells grown in 6-well plates were lysed in 150 µl high salt buffer (see above). Whole lysates were then combined with 150 µl SDS sample buffer and boiled for 5 min before loading on a 7.5% SDS PAGE. MBP-tagged constructs of ORF23 were used in these cotransfection and IP experiments because they yielded higher expression levels and showed less cytotoxicity; the monoclonal anti-MBP-antibody was more effective for IP than the polyclonal ORF23 antibody.

### Apoptosis Assay

The Vibrant Apoptosis Assay (Invitrogen) with Annexin V conjugated to Alexa Fluor 488 was used to evaluate the percentage of apoptotic cells at 24 hr after doxycycline induction of PML IV and PML IV-Δ8AB expressing cell lines. Approximately 5,500 cells per cell line were evaluated by confocal microscopy.

### Statistical Analysis

Student's t tests were performed for all experiments using Graph Pad Prism (version 5.0) statistical software. A p-value of <0.05 was considered statistically significant.

## Supporting Information

Figure S1 Endogenous PML cages are distinct from VZV DNA replication compartments.(A and B) HELF cells were infected with VZV for 24 hr. (A) Viral replication compartments in infected cell nuclei were shown by staining for IE62 (green) and the ORF29 single-strand DNA binding protein (red). These proteins colocalized in viral replication compartments (yellow, merged images). Nuclei were stained with Hoechst (blue). (B) Representative examples of ring-shaped endogenous PML-NBs (green) and VZV replication compartments (IE62, red). Nuclei were stained with Hoechst (blue). The areas in the white squares (i and ii) are shown at higher magnification in the adjacent panels. White arrows indicate the location of ring like PML-NBs. Scale bars are 5 µm.
(1.45 MB TIF)

Figure S2 PML and ORF23 colocalize in VZV-infected melanoma cells.(A) Representative fluorescent microscopy images show PML-NBs (green) and ORF23 capsid protein (red) in melanoma cells infected with VZV and examined at 24 hr post infection. Nuclei were stained with Hoechst (blue). White arrows indicate colocalization of PML-NBs and ORF23 protein. White asterisks indicate infected cell nuclei in which PML-NBs have been completely dispersed. (B) Quantitation of the mean number of PML-NBs in the nuclei of uninfected (N = 180) and infected (N = 180) melanoma cells (mean + SD) examined at 48 hr after VZV infection. (C) Quantitation of the percentage of uninfected or infected cells that contain PML-NBs at 48 hr post infection. Six fields with 30 cells each were analyzed (mean + SD). (D) Western blot analysis of PML protein, IE63 protein, which was used as a marker of VZV infection, and tubulin in whole cell lysates of melanoma cells that were mock infected or infected with VZV (rOka) for 48 hr.
(2.43 MB TIF)

Figure S3 VZV Nucleocapsids (NCs) are associated with endogenous PML-positive fibers. Representative VZV infected HELF cells at 48 hr after infection, as seen after high-pressure freezing, freeze substitution and embedding in LR-white for immunogold-EM. PML protein was identified with a polyclonal (rabbit) anti-PML antibody and Protein-A conjugated with 10 nm gold particles (large arrowheads). Arrows indicate viral NCs. Small arrowheads indicate PML-positive filamentous structures. VZV NCs are associated with PML-positive meshwork (upper left), PML fibers (lower left) or fibrous spherical PML cages (large right panel).
(0.64 MB TIF)

Figure S4 Paracrystalline inclusion bodies of HSV-1 nucleocapsids. HSV-1 infected HELF were fixed and embedded in LR-white for EM analysis at 24 hr after infection (MOI = 0.1). The area in the black square is shown at higher magnification in the right panel and contains a representative paracrystalline cluster of HSV-1 NCs. Note the very regular and dense array of exclusively empty HSV-1 NCs.
(1.48 MB TIF)

Figure S5 Only PML IV promotes the redistribution of ORF23 protein in VZV infected cells. Representative confocal microscopy images show the localization of EGFP-tagged PML-NBs (green) detected in melanoma cells transfected with constructs expressing isoforms I, II, III, IV, V and VI (lower panels); the upper panels show the localization of the isoforms expressing EGFP and the ORF23 protein (red) in transfected cells that were infected with VZV and examined at 48 hr post infection. Nuclei were stained with Hoechst (blue). Only PML IV substantially redistributes ORF23 protein as shown by colocalization in merged images (white arrows).
(3.42 MB TIF)

Figure S6 Immobilization of ORF23 capsid protein by PML IV nuclear bodies in living infected cells. Melanoma cells were transfected with EGFP-PML IV (green) and infected for 48 hr with rOka-RFP-ORF23; this virus expresses the ORF23 capsid protein tagged with RFP. In the pre-bleach panels, live imaging identified EGFP-PML IV bodies (green) that colocalized with RFP-ORF23 protein (red). White arrowheads indicate RFP-ORF23/EGFP-PML IV NBs. The RFP-fluorescence was then selectively bleached (RFP-bleaching) with the 594 nm laser line (bleached area in red); the white circle demarcates the area that was excluded from bleaching and contained two PML IV NBs. The post-bleach panels show imaging done at 15, 30 and 45 min after RFP-bleaching to follow the fate of the bleached and unbleached RFP-ORF23/EGFP-PMLIV complexes. Immobilized ORF23 protein (red) remained confined to the area of PML-NBs (green) up to 45 min after laser bleaching. Scale bar, 5 µm.
(1.03 MB TIF)

Figure S7 PML IV-NBs do not colocalize with VZV replication compartments. Representative fluorescent microscopy images show several EGFP-PML IV bodies (green) and VZV replication compartments (red) that were visualized by staining with antibodies to the ORF29 single stranded DNA binding protein (A) or IE62 (B) or by detection of VZV genomic DNA using in situ hybridization (C) at 48 hr post infection. Nuclei were stained with Hoechst-stain (blue).
(1.87 MB TIF)

Figure S8 Characterization of doxycycline inducible cell lines that express PML IV or PML IV-Δ 8AB.(A) Characterization of doxycycline-inducible PML IV (left panel) and PML IV- Δ 8AB (right panel) melanoma cell lines. Cells were uninduced (-) or induced (+) with doxycycline (5 µg/ml) overnight, fixed and stained for PML (green) and DNA (Hoechst stain, blue). (B) Western blots of whole cell lysates from uninduced (left panel) and induced cells (right panel) were probed with anti-PML polyclonal rabbit antibody and tubulin specific antibody. (C) Annexin V staining for the percentage of apoptotic cells at 24 hr after induction of PML IV (left panel) and PML IV-Δ 8AB (right panel) expressing cell lines. The arrow shows Annexin V-positive cells. Quantification of approximately 5,500 cells of each cell line (11 microscopic fields with 500 cells each) showed no significant difference in the percentage of apoptotic cells between cell lines after induction (p = 0.55).
(2.58 MB TIF)

Figure S9 Ultrastructure of PML IV cages with entrapped VZV nucleocapsids. (A and B) Cells induced to express PML IV and infected with VZV for 48 hr were high-pressure frozen, freeze substituted and embedded in Epoxy resin. PML protein was identified by specific immunogold labeling (PML-15 nm) (A) Comparison of an infected nucleus without PML cages (left panel) and an infected nucleus that contains a large PML cage (right panel, arrow). (B) Comparison of the ultrastructure of PML cages in sections not labeled for PML (left panel) with PML cages identified by PML-specific immunogold labeling (15 nm particles, right panel, arrowheads). PML cages are unequivocally identified only by specific PML immunogold labeling (right panel). (C) Cells induced to express PML IV and infected with VZV for 48 hr were high-pressure frozen, freeze substituted and embedded in LR-White resin for immuno-EM. PML protein was identified by specific immunogold labeling (PML-15 nm). Arrows identify two large PML cages shown at higher magnification in the right panels. Note that the PML cages are densely labeled with PML specific gold particles.
(3.71 MB TIF)

Table S1 Sequences of primers used for the construction of PML deletion mutants.
(0.05 MB DOC)
