# Supplementary figures and images for: Entrapment of Viral Capsids in Nuclear PML Cages Is an Intrinsic Antiviral Host Defense against Varicella-Zoster Virus
Source: PLoS Pathog. 2011 Feb 3;7(2):e1001266. doi: 10.1371/journal.ppat.1001266 (PMC3033373; doi:10.1371/journal.ppat.1001266)

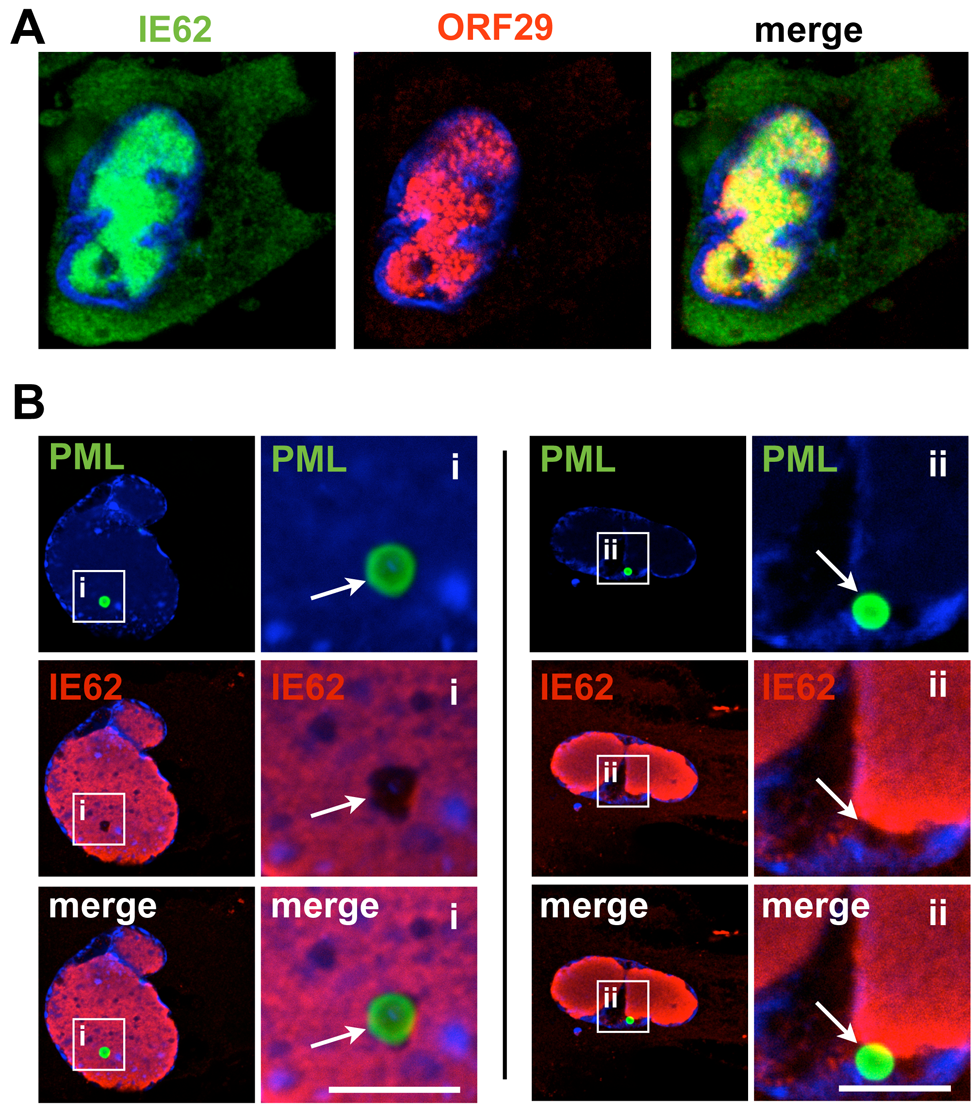

Supplement: Figure S1 — Endogenous PML cages are distinct from VZV DNA replication compartments.(A and B) HELF cells were infected with VZV for 24 hr. (A) Viral replication compartments in infected cell nuclei were shown by staining for IE62 (green) and the ORF29 single-strand DNA binding protein (red). These proteins colocalized in viral replication compartments (yellow, merged images). Nuclei were stained with Hoechst (blue). (B) Representative examples of ring-shaped endogenous PML-NBs (green) and VZV replication compartments (IE62, red). Nuclei were stained with Hoechst (blue). The areas in the white squares (i and ii) are shown at higher magnification in the adjacent panels. White arrows indicate the location of ring like PML-NBs. Scale bars are 5 µm. (1.45 MB TIF) [file ppat.1001266.s001.tif]

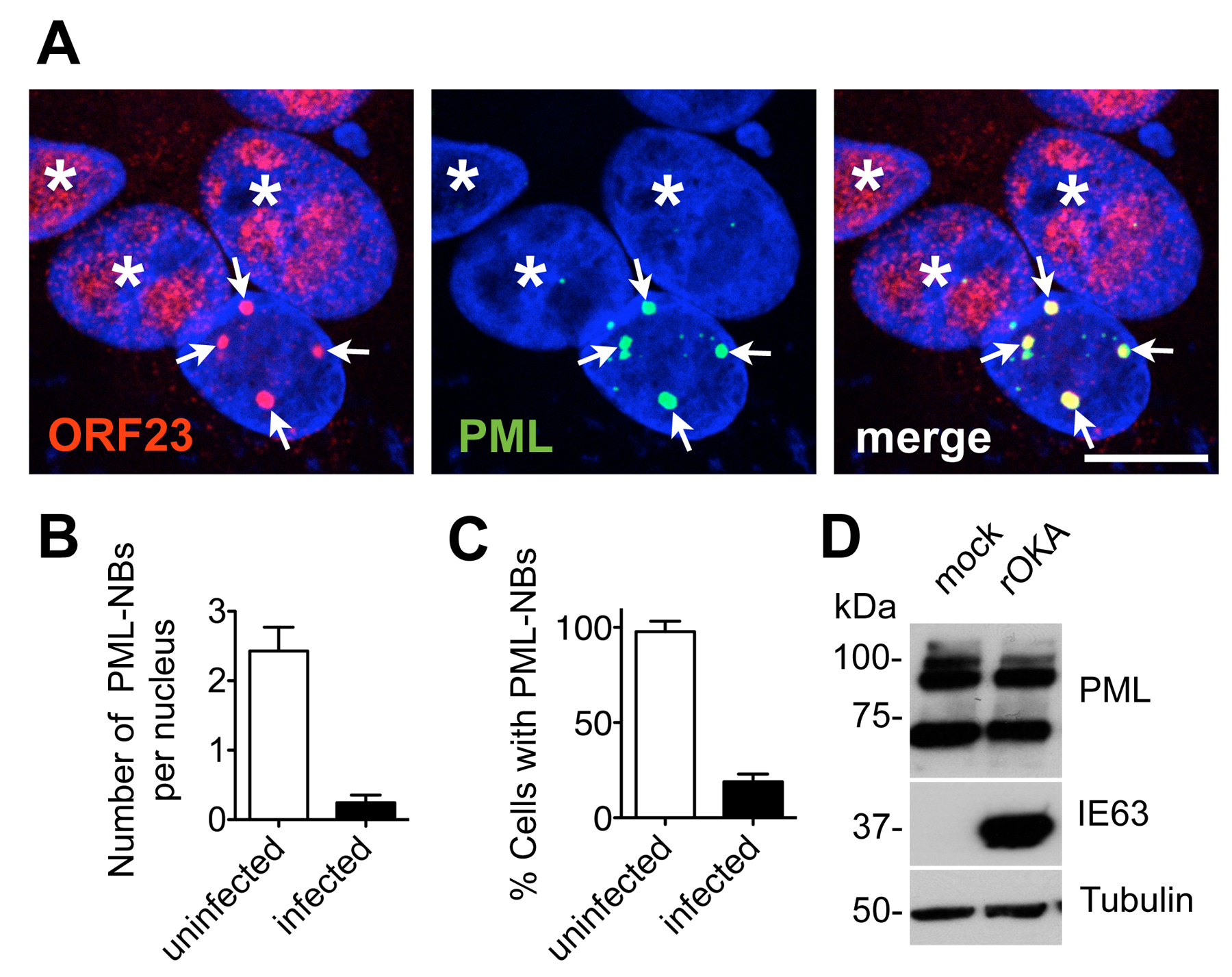

Supplement: Figure S2 — PML and ORF23 colocalize in VZV-infected melanoma cells.(A) Representative fluorescent microscopy images show PML-NBs (green) and ORF23 capsid protein (red) in melanoma cells infected with VZV and examined at 24 hr post infection. Nuclei were stained with Hoechst (blue). White arrows indicate colocalization of PML-NBs and ORF23 protein. White asterisks indicate infected cell nuclei in which PML-NBs have been completely dispersed. (B) Quantitation of the mean number of PML-NBs in the nuclei of uninfected (N = 180) and infected (N = 180) melanoma cells (mean + SD) examined at 48 hr after VZV infection. (C) Quantitation of the percentage of uninfected or infected cells that contain PML-NBs at 48 hr post infection. Six fields with 30 cells each were analyzed (mean + SD). (D) Western blot analysis of PML protein, IE63 protein, which was used as a marker of VZV infection, and tubulin in whole cell lysates of melanoma cells that were mock infected or infected with VZV (rOka) for 48 hr. (2.43 MB TIF) [file ppat.1001266.s002.tif]

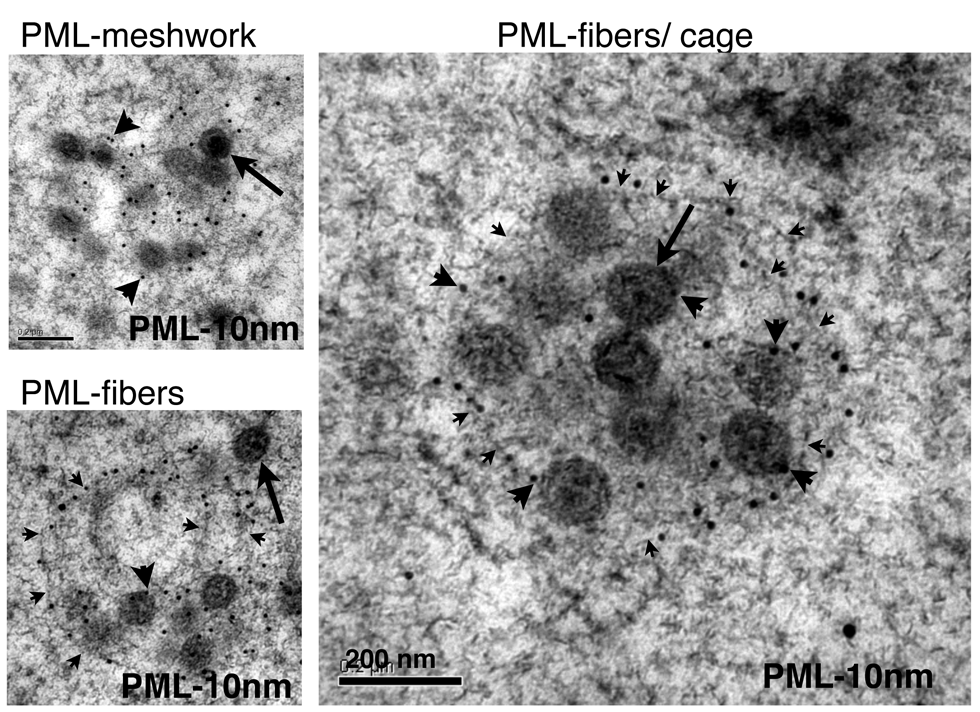

Supplement: Figure S3 — VZV Nucleocapsids (NCs) are associated with endogenous PML-positive fibers. Representative VZV infected HELF cells at 48 hr after infection, as seen after high-pressure freezing, freeze substitution and embedding in LR-white for immunogold-EM. PML protein was identified with a polyclonal (rabbit) anti-PML antibody and Protein-A conjugated with 10 nm gold particles (large arrowheads). Arrows indicate viral NCs. Small arrowheads indicate PML-positive filamentous structures. VZV NCs are associated with PML-positive meshwork (upper left), PML fibers (lower left) or fibrous spherical PML cages (large right panel). (0.64 MB TIF) [file ppat.1001266.s003.tif]

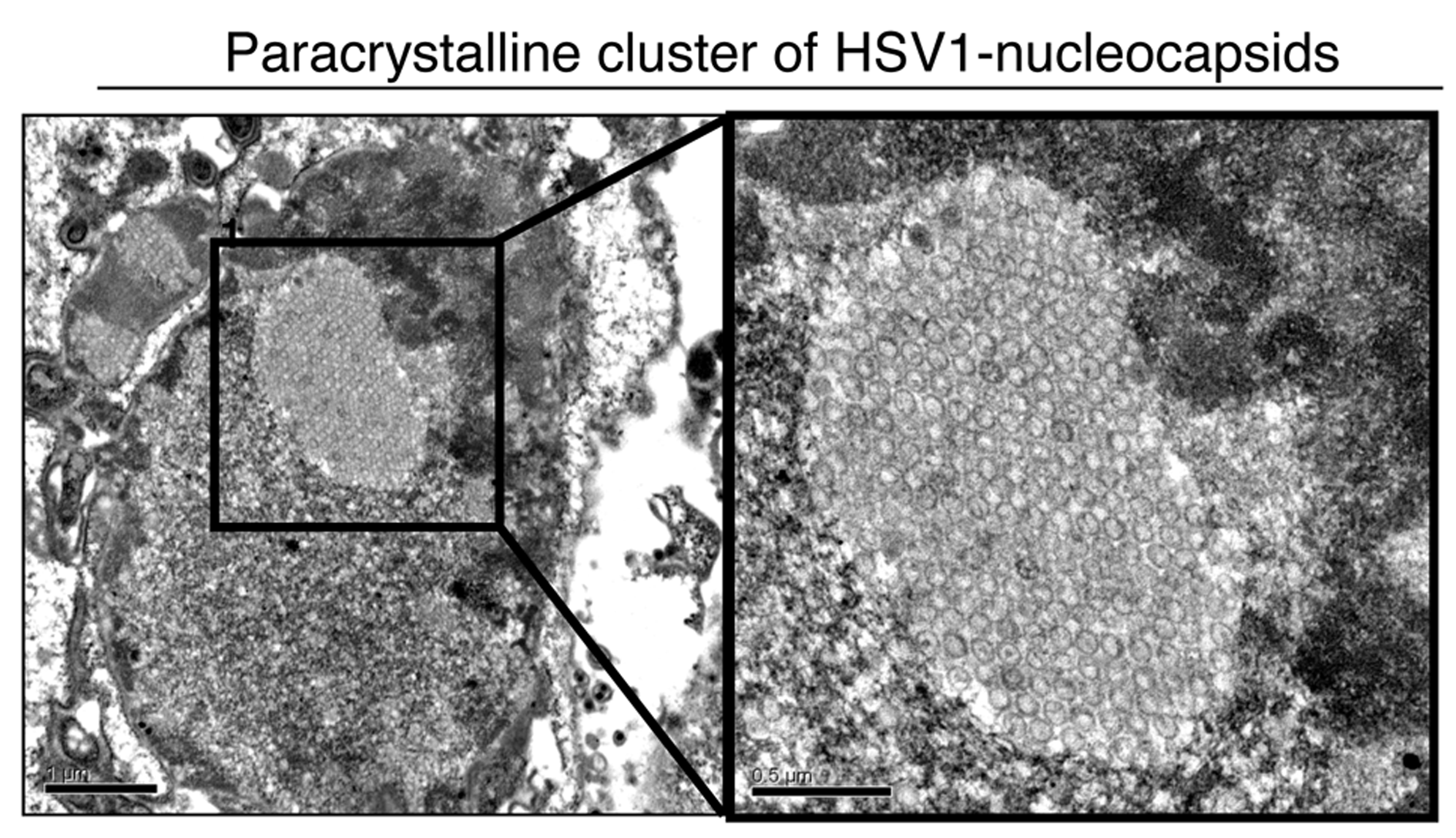

Supplement: Figure S4 — Paracrystalline inclusion bodies of HSV-1 nucleocapsids. HSV-1 infected HELF were fixed and embedded in LR-white for EM analysis at 24 hr after infection (MOI = 0.1). The area in the black square is shown at higher magnification in the right panel and contains a representative paracrystalline cluster of HSV-1 NCs. Note the very regular and dense array of exclusively empty HSV-1 NCs. (1.48 MB TIF) [file ppat.1001266.s004.tif]

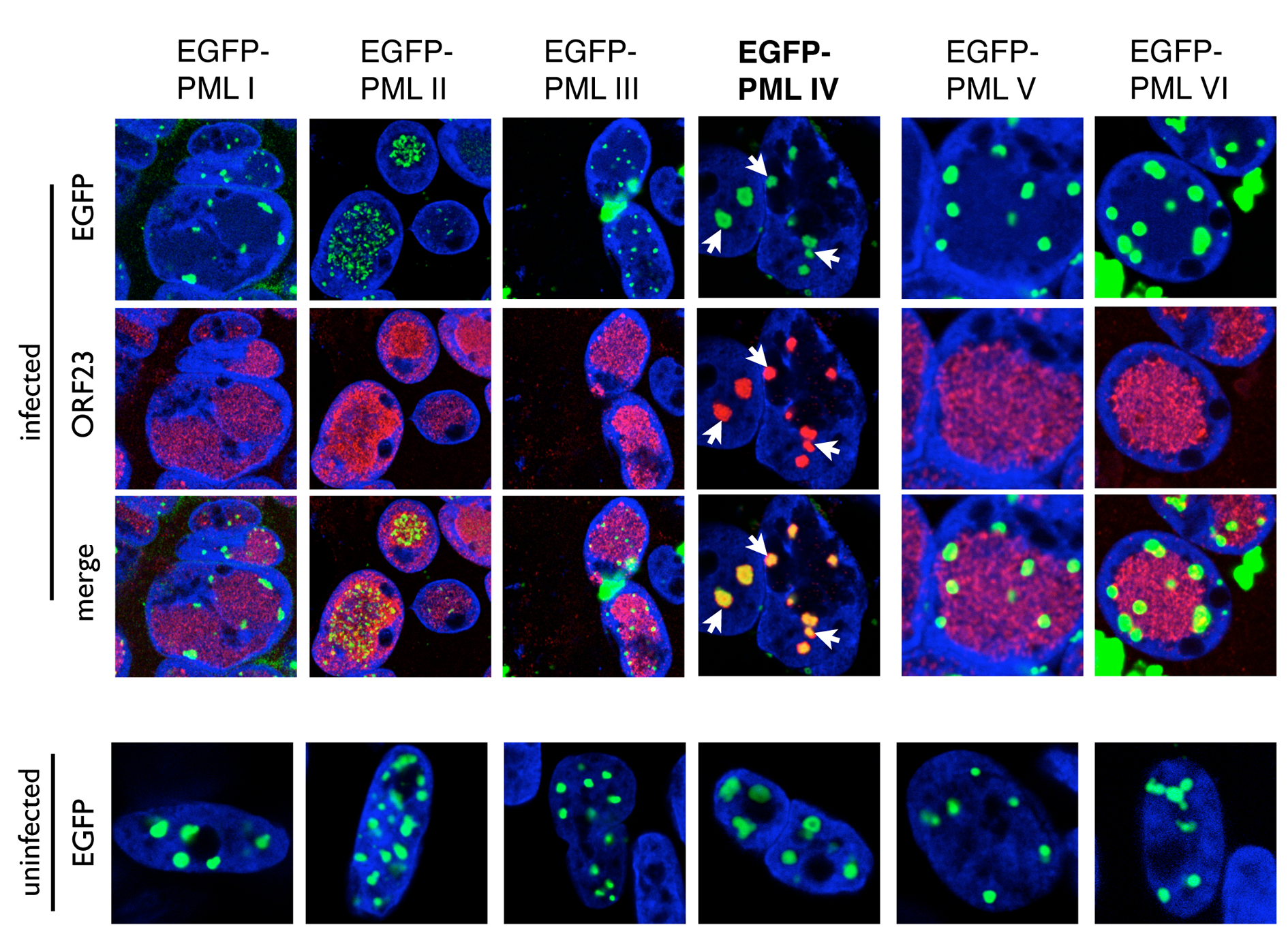

Supplement: Figure S5 — Only PML IV promotes the redistribution of ORF23 protein in VZV infected cells. Representative confocal microscopy images show the localization of EGFP-tagged PML-NBs (green) detected in melanoma cells transfected with constructs expressing isoforms I, II, III, IV, V and VI (lower panels); the upper panels show the localization of the isoforms expressing EGFP and the ORF23 protein (red) in transfected cells that were infected with VZV and examined at 48 hr post infection. Nuclei were stained with Hoechst (blue). Only PML IV substantially redistributes ORF23 protein as shown by colocalization in merged images (white arrows). (3.42 MB TIF) [file ppat.1001266.s005.tif]

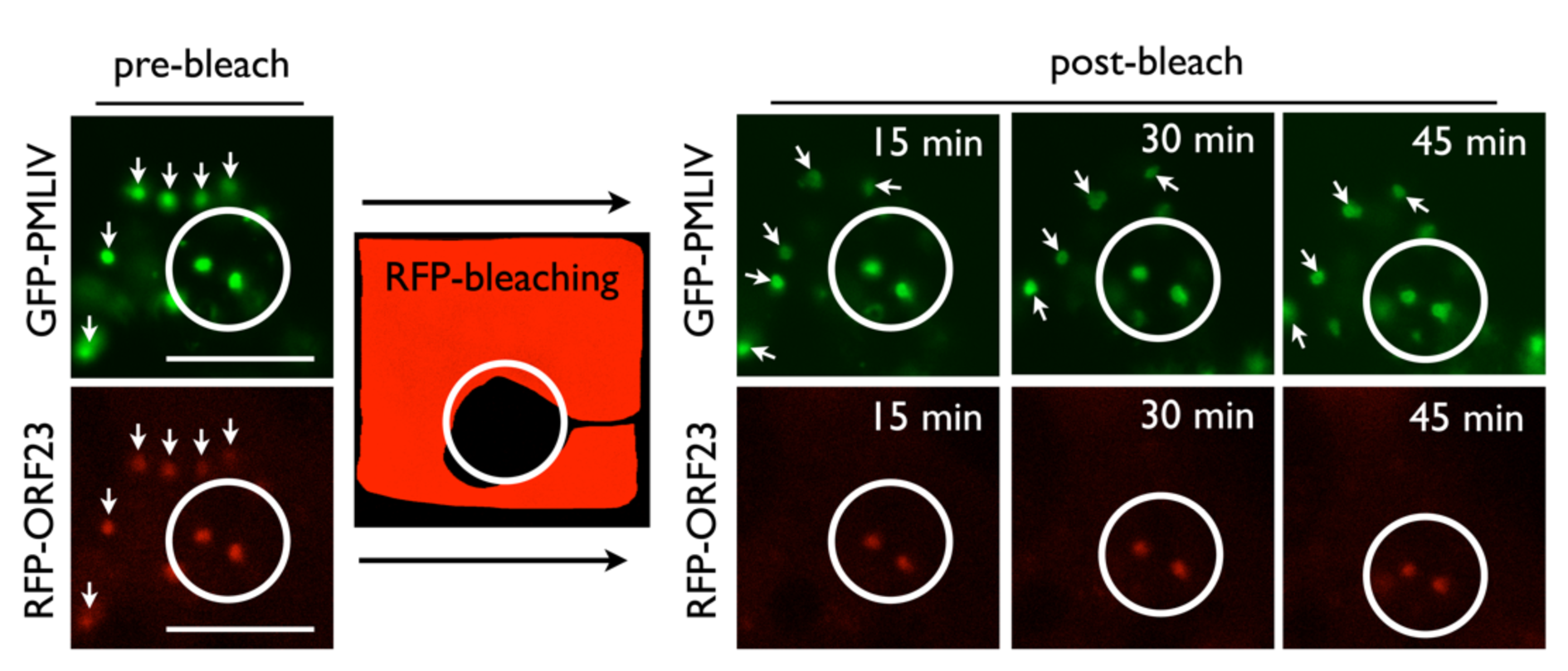

Supplement: Figure S6 — Immobilization of ORF23 capsid protein by PML IV nuclear bodies in living infected cells. Melanoma cells were transfected with EGFP-PML IV (green) and infected for 48 hr with rOka-RFP-ORF23; this virus expresses the ORF23 capsid protein tagged with RFP. In the pre-bleach panels, live imaging identified EGFP-PML IV bodies (green) that colocalized with RFP-ORF23 protein (red). White arrowheads indicate RFP-ORF23/EGFP-PML IV NBs. The RFP-fluorescence was then selectively bleached (RFP-bleaching) with the 594 nm laser line (bleached area in red); the white circle demarcates the area that was excluded from bleaching and contained two PML IV NBs. The post-bleach panels show imaging done at 15, 30 and 45 min after RFP-bleaching to follow the fate of the bleached and unbleached RFP-ORF23/EGFP-PMLIV complexes. Immobilized ORF23 protein (red) remained confined to the area of PML-NBs (green) up to 45 min after laser bleaching. Scale bar, 5 µm. (1.03 MB TIF) [file ppat.1001266.s006.tif]

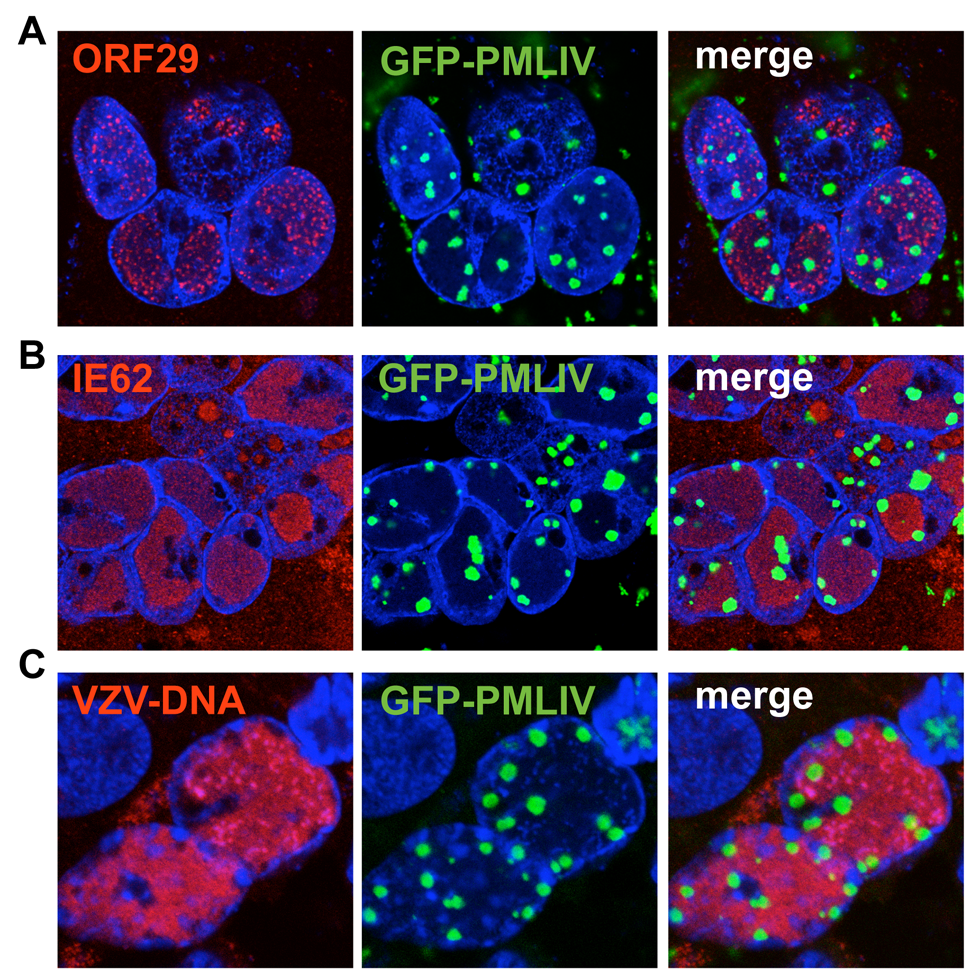

Supplement: Figure S7 — PML IV-NBs do not colocalize with VZV replication compartments. Representative fluorescent microscopy images show several EGFP-PML IV bodies (green) and VZV replication compartments (red) that were visualized by staining with antibodies to the ORF29 single stranded DNA binding protein (A) or IE62 (B) or by detection of VZV genomic DNA using in situ hybridization (C) at 48 hr post infection. Nuclei were stained with Hoechst-stain (blue). (1.87 MB TIF) [file ppat.1001266.s007.tif]

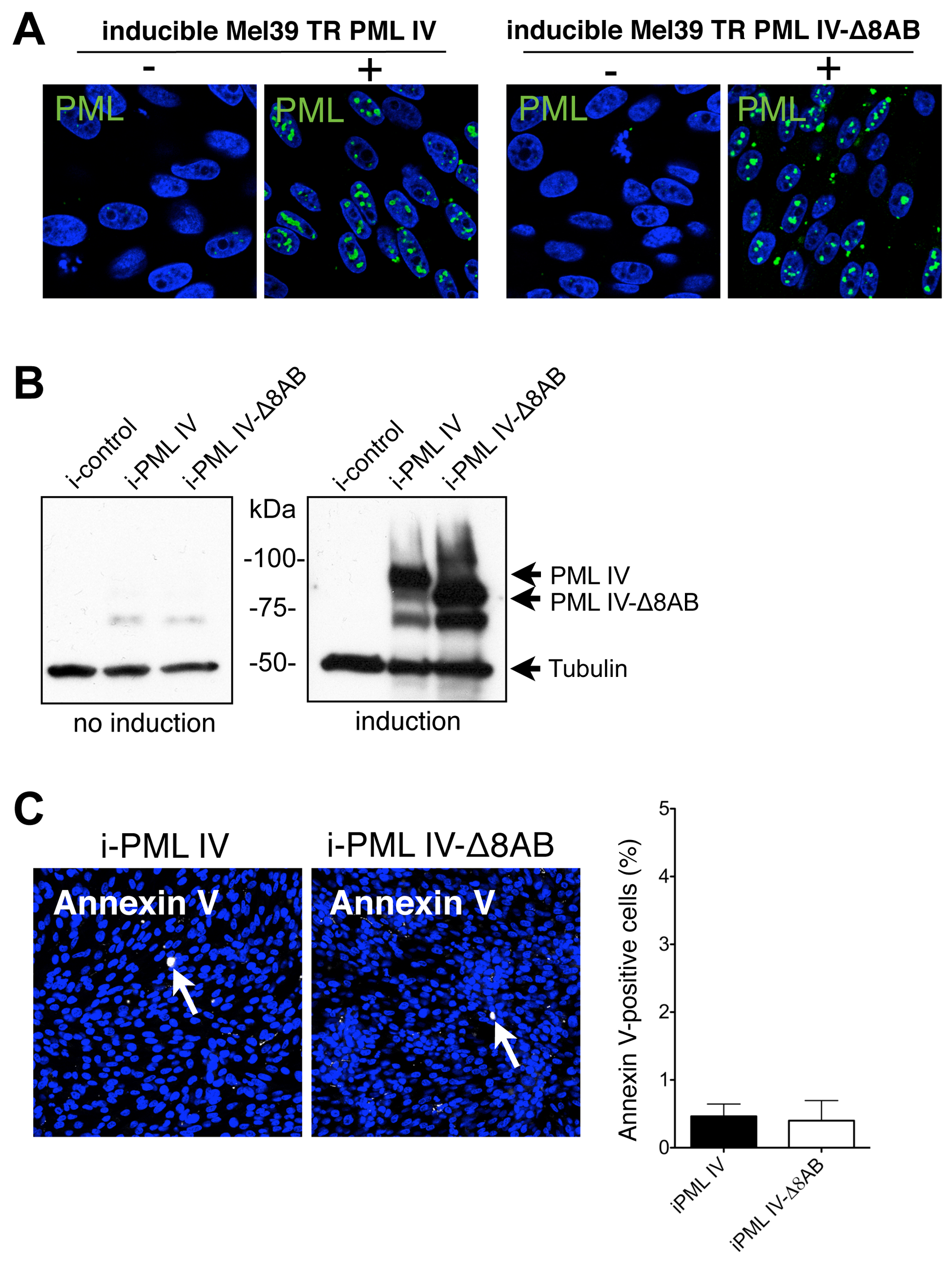

Supplement: Figure S8 — Characterization of doxycycline inducible cell lines that express PML IV or PML IV-Δ 8AB.(A) Characterization of doxycycline-inducible PML IV (left panel) and PML IV- Δ 8AB (right panel) melanoma cell lines. Cells were uninduced (-) or induced (+) with doxycycline (5 µg/ml) overnight, fixed and stained for PML (green) and DNA (Hoechst stain, blue). (B) Western blots of whole cell lysates from uninduced (left panel) and induced cells (right panel) were probed with anti-PML polyclonal rabbit antibody and tubulin specific antibody. (C) Annexin V staining for the percentage of apoptotic cells at 24 hr after induction of PML IV (left panel) and PML IV-Δ 8AB (right panel) expressing cell lines. The arrow shows Annexin V-positive cells. Quantification of approximately 5,500 cells of each cell line (11 microscopic fields with 500 cells each) showed no significant difference in the percentage of apoptotic cells between cell lines after induction (p = 0.55). (2.58 MB TIF) [file ppat.1001266.s008.tif]

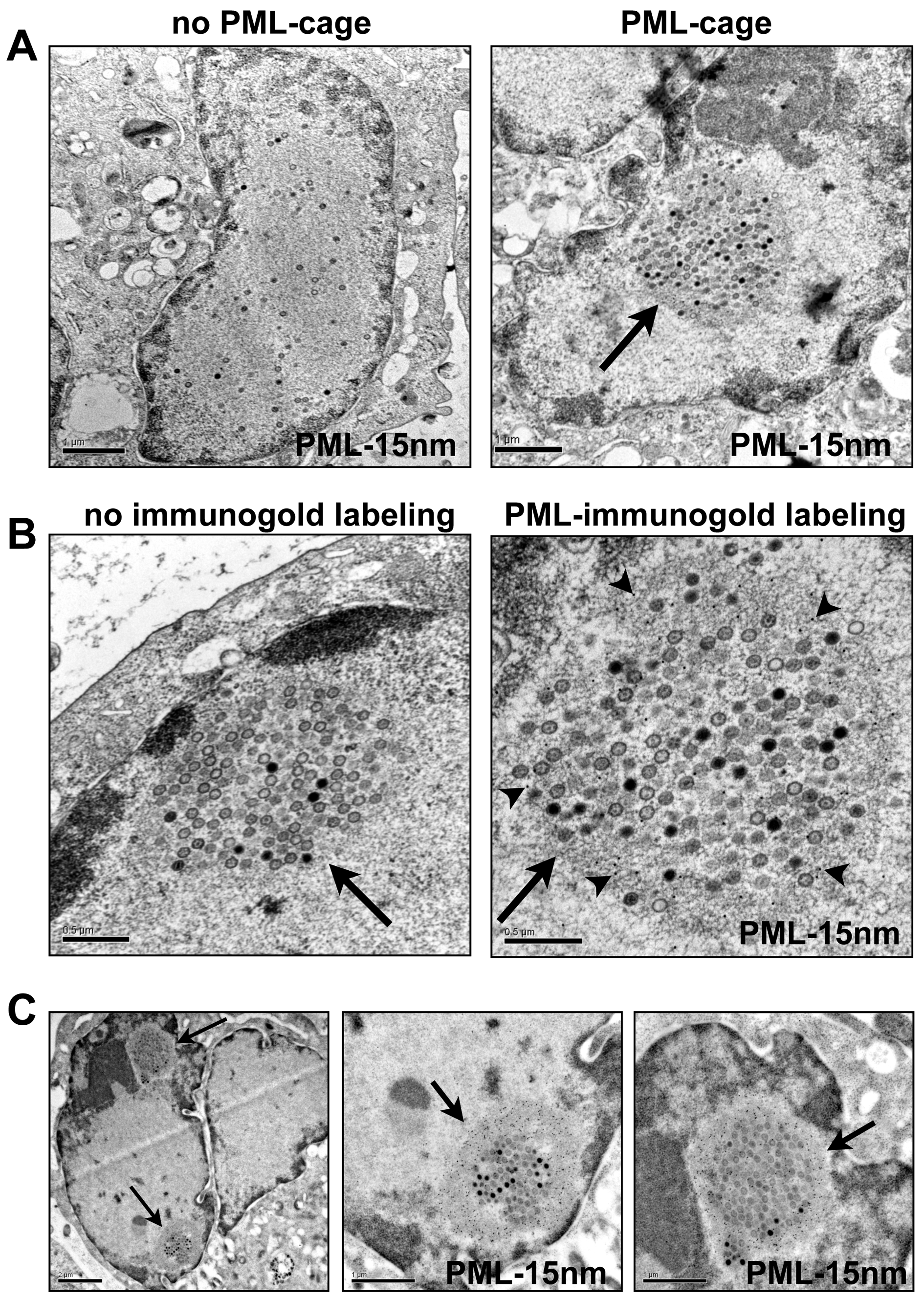

Supplement: Figure S9 — Ultrastructure of PML IV cages with entrapped VZV nucleocapsids. (A and B) Cells induced to express PML IV and infected with VZV for 48 hr were high-pressure frozen, freeze substituted and embedded in Epoxy resin. PML protein was identified by specific immunogold labeling (PML-15 nm) (A) Comparison of an infected nucleus without PML cages (left panel) and an infected nucleus that contains a large PML cage (right panel, arrow). (B) Comparison of the ultrastructure of PML cages in sections not labeled for PML (left panel) with PML cages identified by PML-specific immunogold labeling (15 nm particles, right panel, arrowheads). PML cages are unequivocally identified only by specific PML immunogold labeling (right panel). (C) Cells induced to express PML IV and infected with VZV for 48 hr were high-pressure frozen, freeze substituted and embedded in LR-White resin for immuno-EM. PML protein was identified by specific immunogold labeling (PML-15 nm). Arrows identify two large PML cages shown at higher magnification in the right panels. Note that the PML cages are densely labeled with PML specific gold particles. (3.71 MB TIF) [file ppat.1001266.s009.tif]
