# Supplementary material for: Entrapment of Viral Capsids in Nuclear PML Cages Is an Intrinsic Antiviral Host Defense against Varicella-Zoster Virus
Source: PLoS Pathog. 2011 Feb 3;7(2):e1001266. doi: 10.1371/journal.ppat.1001266 (PMC3033373; doi:10.1371/journal.ppat.1001266)
Supplement: Table S1 — Sequences of primers used for the construction of PML deletion mutants. (0.05 MB DOC) [file ppat.1001266.s010.doc]

**Table S1 Sequences of primers used for the construction of PML deletion mutants.**

| **Primer** | **Sequence (5’ to 3’)** | **Information** |
| --- | --- | --- |
| PML I∆9 5’A | GCTGGTGCAGAGGATGAAGTGCTAC | Anneals upstream of a Mlu I site. |
| PML I∆9 5’B | TTCATTGTCAATCTTGAGGTCA AAGAAAAC | 5’ end anneals at the last codon of exon 8a in PML I. |
| PML I∆9 3’A | TGAGAGGAGGGGGTGACCAG | 5’ end anneals at the stop codon following exon 9. |
| PML I∆9 3’B | TTTCGCCCTTTGACGTTGGAG | Anneals downstream of a MluI site. |
| PMLIV∆8b 5’A | GCTGGTGCAGAGGATGAAGTGCTAC | Anneals upstream of a BbvCI site. |
| PMLIV∆8ab 5’A | AGGAGCTGGACGCCATGAC | Anneals upstream of a BbvCI site. |
| PMLIV∆8ab 5’B | CGAGTTTTCGGCATCTGAGTC | 5’ end anneals at the last codon of exon 7a in PML IV. |
| PML IV 3’A | TAGTCTCTGAGTCCCAAAAAGAAGTGC | 5’ end anneals at the stop codon following exon 8b |
| PMLIV3’B | ATCCTCATCCTGTCTCTTGATCGA | Anneals downstream of a MluI site. |
| 5’PML IV | GGATCCGCCACCATGGAGCCTGCACCCGCCCGATCTCC | Inserts BamHI site upstream of PML IV start codon. |
| 5’EGFP-PML IV | GGATCCGCCACCATGGTGAGCAAGGGCGAGGAGC | Inserts BamHI site upstream of EGFP PML IV start codon (italics). |
| 3’PML IV | GAATTCTCACTAAATTAGAAAGGGGTGGGGGTAGC | Inserts EcoRI site downstream of PML IV stop codon. |
| 3’PML IV∆8ab | GAATTCTCACTACGAGTTTTCGGCATCTGAGTCTTCC | Inserts EcoRI site downstream of PML IV∆8ab stop codon. |
|  |  |  |
